# The early excitatory action of striatal cholinergic-GABAergic microcircuits conditions the subsequent GABA inhibitory shift

**DOI:** 10.1038/s42003-023-05068-7

**Published:** 2023-07-14

**Authors:** Natalia Lozovaya, Sanaz Eftekhari, Constance Hammond

**Affiliations:** 1 B&A Therapeutics, Marseille, France; 2 Neurochlore, Marseille, France

**Keywords:** Neurophysiology, Synaptic development

## Abstract

Cholinergic interneurons of the striatum play a role in action selection and associative learning by activating local GABAergic inhibitory microcircuits. We investigated whether cholinergic-GABAergic microcircuits function differently and fulfill a different role during early postnatal development, when GABA_A_ actions are not inhibitory and mice pups do not walk. We focused our study mainly on dual cholinergic/GABAergic interneurons (CGINs). We report that morphological and intrinsic electrophysiological properties of CGINs rapidly develop during the first post-natal week. At this stage, CGINs are excited by the activation of GABA_A_ receptors or GABAergic synaptic inputs, respond to cortical stimulation by a long excitation and are linked by polysynaptic excitations. All these excitations are replaced by inhibitions at P12-P15. Early chronic treatment with the NKCC1 antagonist bumetanide to evoke premature GABAergic inhibitions from P4 to P8, prevented the GABA polarity shift and corticostriatal pause response at control postnatal days. We propose that early excitatory cholinergic-GABAergic microcircuits are instrumental in the maturation of GABAergic inhibition.

## Abstract (web summary)

Electrophysiological recordings reveal that early excitatory cholinergic-GABAergic microcircuits are important for the maturation of GABAergic inhibition in the mouse striatum.

## Introduction

Cholinergic interneurons constitute the main source of acetylcholine in the striatum and are the only local excitatory neurons via activation of nicotinic receptors^1^. However, cholinergic interneurons do not represent a homogeneous population^2,3^, and comprise a subpopulation of cholinergic/GABAergic interneurons (CGINs) that co-express cholinergic and GABAergic markers^4^. In spite of this heterogeneity, all cholinergic interneurons share a characteristic giant soma with 3-6 thick and sparsely spiny, primary dendrites that branch modestly and extend in a radial pattern^5^. Cholinergic interneurons spontaneously fire and their activity is characterized by a prominent rectification due to the hyperpolarization-activated cationic current (I_h_)^6^. Despite their small number, cholinergic interneurons play a key role in the modulation of striatal GABAergic microcircuits, thanks to their extensive local connections (they are estimated to have 500000 axonal varicosities each^5^ and synchronizing capacity. They connect cortical or thalamic afferents^7^ to output neurons of the striatum via GABAergic interneurons^8–12^, and are interconnected by a strong, prevalent, local, polysynaptic GABAergic network that promotes their synchrony^13,14^. The main cholinergic interneurons signature is a pause (inhibitory) response to cortical or thalamic stimulation in vitro^15^ or in vivo in response to salient or reward prediction-related stimuli after conditioning in rodents^13^ and primates^16^. These and other observations suggest that cholinergic interneurons play important roles in action selection^17^ and in signaling changes in reward contingencies to enable behavioral flexibility^18,19^.

The maturation of striatal cholinergic interneurons has not yet been thoroughly investigated^20^. This is of particular importance in view of the major changes occurring during development and notably the shift of GABA_A_R current polarity from depolarizing to hyperpolarizing (GABA polarity shift), due to a reduction of [Cl^-^]_i_ levels reported in many animal species and brain structures^21^. Interestingly, cholinergic interneurons are the earliest born neurons in striatal neurogenesis^22,23^. In mice, most striatal cholinergic interneurons are generated before E14.5, with over 50% of them being generated before E12.5^24^. This raises the possibility of a particular role of cholinergic interneurons during striatal development, different from their adult role as critical nodes in striatal synaptic computation^25,26^.

In this aim, we first determined the development of intrinsic properties of cholinergic interneurons and of cholinergic-GABAergic microcircuits. We focused this study mainly on dual cholinergic/GABAergic interneurons (CGINs, see methods) of the dorsal striatum, from the late embryonic period (E16) to the second postnatal week (P15). We show that most CGINs intrinsic morphological, electrophysiological parameters and synaptic connections are developed by the end of the first postnatal week. In striking contrast, GABA polarity matures later around P10-P12, leaving a few days during which CGINs respond to cortical stimulation by a GABAergic excitation instead of the classical pause response and are interconnected by an excitatory GABAergic polysynaptic pathway. Chronic blockade of the chloride importer Na^+^-K^+^−2Cl^-^ (NKCC1) by the specific antagonist bumetanide between P4 and P8 prevented the subsequent GABA polarity shift and the development of the cortico-striatal inhibitory pause response. We suggest that early excitatory GABAergic activity in cholinergic-GABAergic microcircuits is instrumental in the maturation of striatal GABAergic inhibition.

## Results

**Fig. 1:**
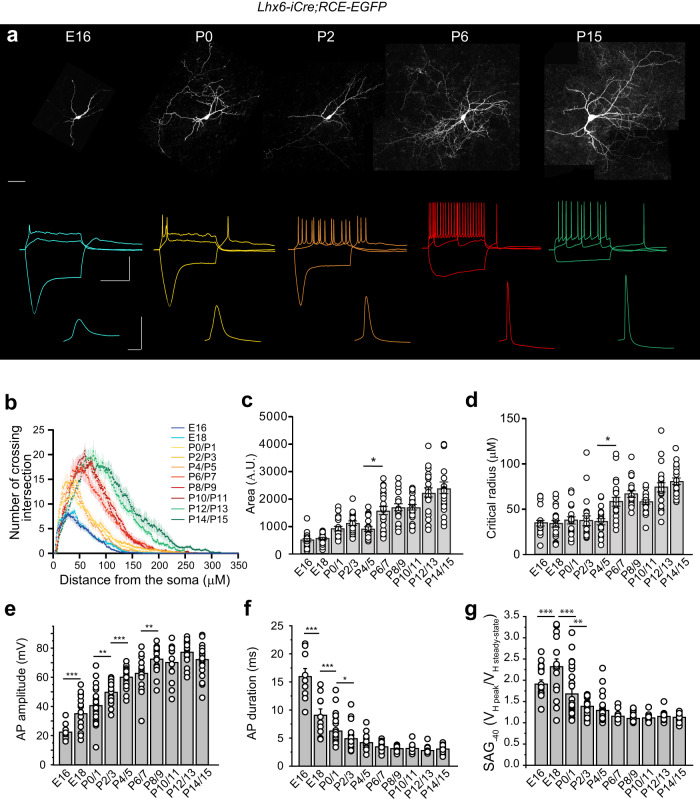
Development of CGINs intrinsic properties from E16 to P15: a summary. **a** (top) Representative biocytin-filled EGFP^+^ cholinergic interneurons (CGINs) and (bottom) representative voltage responses to hyperpolarizing (−40 pA (E16), −50 pA (P0), −100 pA (P2, P6, P15)) and depolarizing current pulses and corresponding action potential waveforms at expanded time scale. Scale bars: 50 μm; 50 mV and 1 s; 50 mV, 25 ms. **b** Mean Sholl profiles. **c** Mean areas under the Sholl curves. **d** Mean critical radius (E16: *n* = 21 (*N* = 5); E18: *n* = 21 (*N* = 3); P0/1: *n* = 20 (*N* = 6); P2/3: *n* = 20 (*N* = 7); P4/5: *n* = 20 (*N* = 13); P6/7: *n* = 21 (*N* = 4); P8/9: *n* = 20 (*N* = 5); P10/11: *n* = 21 (*N* = 12); P12/13: *n* = 21 (*N* = 5); P14/15: *n* = 20 (*N* = 7). **e**, **f** Mean peak amplitudes (E16: *n* = 12 (*N* = 7); E18: *n* = 15 (*N* = 6); P0/1: *n* = 29 (*N* = 8); P2/3: *n* = 22 (*N* = 7); P4/5: *n* = 20 (*N* = 6); P6/7: *n* = 17 (*N* = 4); P8/9: *n* = 17 (*N* = 4); P10/11: *n* = 12 (*N* = 4); P12/13: *n* = 20 (*N* = 4); P14/15: *n* = 22 (*N* = 6) and mean half-amplitude durations (E16: *n* = 9 (*N* = 7); E18: *n* = 10 (*N* = 6); P0/1: *n* = 26 (*N* = 8); P2/3: *n* = 22 (*N* = 7); P4/5: *n* = 20 (*N* = 6); P6/7: *n* = 17 (*N* = 4); P8/9: *n* = 17 (*N* = 4); P10/11: *n* = 12 (*N* = 4); P12/13: *n* = 20 (*N* = 4); P14/15: *n* = 20 (*N* = 6) of evoked action potentials (AP). **g** Mean amplitudes of the sag in response to a −40 pA hyperpolarizing pulse (E16: *n* = 19 (*N* = 7); E18: *n* = 15 (*N* = 6); P0/1: *n* = 25 (*N* = 8); P2/3: *n* = 18 (*N* = 7); P4/5: *n* = 25 (*N* = 6); P6/7: *n* = 17 (*N* = 4); P8/9: *n* = 17 (*N* = 4); P10/11: *n* = 12 (*N* = 4); P12/13: *n* = 17 (*N* = 4); P14/15: *n* = 22 (*N* = 6)). All means ± s.e.m (Supplementary Table 2). *n* = number of cells, *N* = number of mice.

We first determined the time window extending from E16 (when ChAT+ interneurons can be reliably identified) and P14 when all pups have acquired quadruped ambulation (see Methods, Supplementary Fig. 1, 2, Supplementary Table 1, Supplementary Note). We studied, during this time window (E16-P15), the properties and possible developmental role of cholinergic interneurons, in particular CGINs (Fig. 1), and cholinergic-GABAergic polysynaptic microcircuits of the dorsal striatum. Throughout the text, cholinergic interneurons are ChAT^+^ EGFP^-^ and ChAT^+^ EGFP^+^ interneurons while cholinergic/GABAergic interneurons (CGINs) are only ChAT^+^ EGFP^+^ interneurons, previously shown to co-express GABAergic markers^4^. We recorded 557 CGINs and 140 cholinergic interneurons located in the dorsal striatum, from 145 Lhx6-EGFP mice and 29 ChAT-ChR2-EYFP mice, respectively.)

### CGINs intrinsic properties

Figure 1a shows an overall view of the typical development of CGINs morphological and physiological intrinsic properties. Sholl analysis of the density profiles of branches of 3D-reconstructed, ChAT^+^ EGFP^+^, biocytin-filled CGINs (Fig. 1a top) as a function of distance from the soma (Supplementary Fig. 3a, b), showed a critical step at P6/7 for the area under the curve and the critical radius (Fig. 1b–d, Supplementary Table 2). The mean area under the Sholl curve, critical values (peak of maximum branch density), total dendritic lengths, number of dendritic nodes, cell ending radius, all quickly and exponentially developed from E16 to P6/P7 (Fig. 1c, Supplementary Figs. 3c–f, 5a, c–f, p Supplementary Tables 3, 6) whereas the number of dendritic trees (trunks, 1 to 9, mean 4 ± 1) was already definitive at E16 (Supplementary Table 3). Except for cell ending radius, all other parameters showed a change of speed of development before the end of the first postnatal week (Supplementary Fig. 5a–f, p).

Sodium spikes were generated by half of E16 CGINs (*n* = 11/21 cells, *N* = 5 mice) but with immature characteristics (Fig. 1a). Spike amplitude, half-width duration and threshold, maximal spike frequency, after spike hyperpolarization (AHP) amplitude, and “sag” displayed back towards baseline during a hyperpolarizing current pulse caused by activation of the hyperpolarization-activated, cyclic nucleotide-gated, non-specific cation (HCN) current (I_h_) (Fig. 1a, e–g and Supplementary Figs. 4a–c, 6b), all exponentially developed from E18 to P4/7 (Supplementary Figs. 5h–n, q), (Supplementary Tables 2, 4, 5, 6). After P4/7, these parameters reached a steady state or begun a slower progression. In contrast, membrane resistance (Rm) regularly and exponentially decreased until P15 (Supplementary Figs. 4c, 5g, q). This is to be correlated to the continuous increase of total dendritic length, critical radius and ending radius during the same period (Fig. 1d, Supplementary Fig. 3c, e). All these results suggested a rapid development of morphological and physiological CGINs parameters from E16/18 to the end of the first postnatal week, with a decisive change before P7. Physiological parameters also suggested that CGINs develop as a homogeneous population of striatal cholinergic interneurons (Supplementary Fig. 4d).

Intrinsic firing of cholinergic interneurons of the striatum is dependent on subthreshold currents such as the hyperpolarization-activated cyclic nucleotide-gated cationic current (I_h_) and the persistent sodium current (I_NaP_)^6^. I_h_ was already present at E16 as attested by the presence of a large amplitude sag in current clamp recordings and of the large amplitude, ZD7288-sensitive, slowly developing inward current in voltage clamp recordings (Fig. 1a, g, Supplementary Fig. 6a, f). The sag evoked by current steps to −40 or −100 mV, exponentially decreased from E18/P0 to P6/7 where it reached a steady state (Supplementary Figs. 6a–c, 5m, n, and Supplementary Tables 2, 5). During the same period I_h_ exponentially increased and reached a steady state at P6/7 (Supplementary Figs. 6d, 5o, Supplementary Table 5). The decrease of sag amplitude though I_h_ intensity continuously increased, resulted from the continuous decrease of membrane resistance and the significant decrease of I_h_ current density from P4/5 (Supplementary Fig. 6e, and Supplementary Table 5). Specific blockade of I_h_ by ZD7288, totally blocked the sag, produced a significant hyperpolarization of the membrane resting potential (*V*_Rest_), a delay of spike onset and a decrease of the maximal firing frequency of CGINs at P4/5 and P14/15 (Supplementary Fig. 6f–k and Supplementary Table 5), showing the crucial early role of I_h_ on CGINs excitability at P5 and P15.

The persistent sodium current (*I*_NaP_) also played a role on CGINs activity but later than I_h_. In response to a depolarizing voltage ramp from −70 mV, an inward current that had the characteristics of I_NaP_ (activated at subthreshold potentials, sensitive to nanomolar doses of TTX) was recorded at P5 but with a small amplitude (−29 ± 3 pA, *n* = 9 cells, *N* = 4 mice). It significantly increased at P7 (−77 ± 17 pA, *n* = 7 cells, *N* = 3 mice) and stayed stable until P14/15 (Supplementary Fig. 7a, b). Bath application of TTX at a low dose to preferentially inhibit I_NaP_, decreased evoked firing frequency and blocked rebound spikes at P7 (Supplementary Fig. 7a, c, d and Supplementary Table 7).

### The developmental shift of GABA polarity

The switch of GABA action from depolarizing to hyperpolarizing mode is a critical event for early brain circuits’ development and their orchestrated activity-dependent formation^21^. In several brain structures, during fetal and postnatal periods, GABA is the primary depolarizing/excitatory neurotransmitter with a transient perinatal shift to inhibition due to a reduction in the intracellular chloride concentration. Later in development, during the second postnatal week, persistent switch to inhibitory action occurs, and is a signature of networks maturation.

**Fig. 2:**
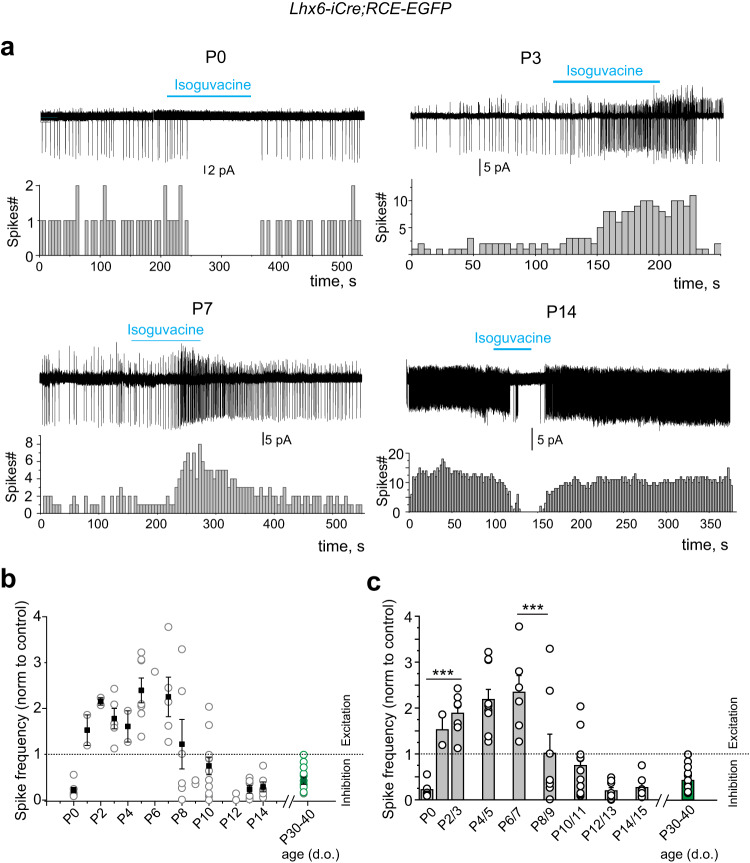
Developmental shift of GABA polarity from excitatory to inhibitory. **a** Representative cell-attached recordings (top) and corresponding frequency histograms (bottom) of ongoing spike activity before, during and after isoguvacine (10 μM) application at the indicated ages. **b**, **c** Mean values of isoguvacine maximal effects on spike frequency (P30–40 data from ref. ^4^), (P0: *n* = 7 (*N* = 3); P2/3: *n* = 7 (*N* = 3); P4/5: *n* = 10 (*N* = 5); P6/7: *n* = 6 (*N* = 4); P8/9: *n* = 8 (*N* = 4); P10/11: *n* = 11 (*N* = 4); P12/13: *n* = 8 (*N* = 3); P14/15: *n* = 7 (*N* = 3) CGINs; P30/40: *n* = 13 (*N* = 7) CGINs). All means ± s.e.m (Supplementary Table 8). *n* = number of cells, *N* = number of mice.

To test CGINs responses to GABA during postnatal development we compared the effects of the GABA_A_R agonist isoguvacine on CGINs activity in non-invasive, cell-attached, patch-clamp recordings. At P0, focal application of isoguvacine decreased the frequency of ongoing spike activity, whereas from P2 to P7, it increased it. At P8 isoguvacine had no effect on spike frequency and began to be inhibitory at P10 and stayed inhibitory thereafter (Fig. 2, and Supplementary Table 8). CGINs response to GABA_A_R activation followed the classical pattern observed in other brain regions.

### The developmental role of GABA_A_ R-mediated activity

**Fig. 3:**
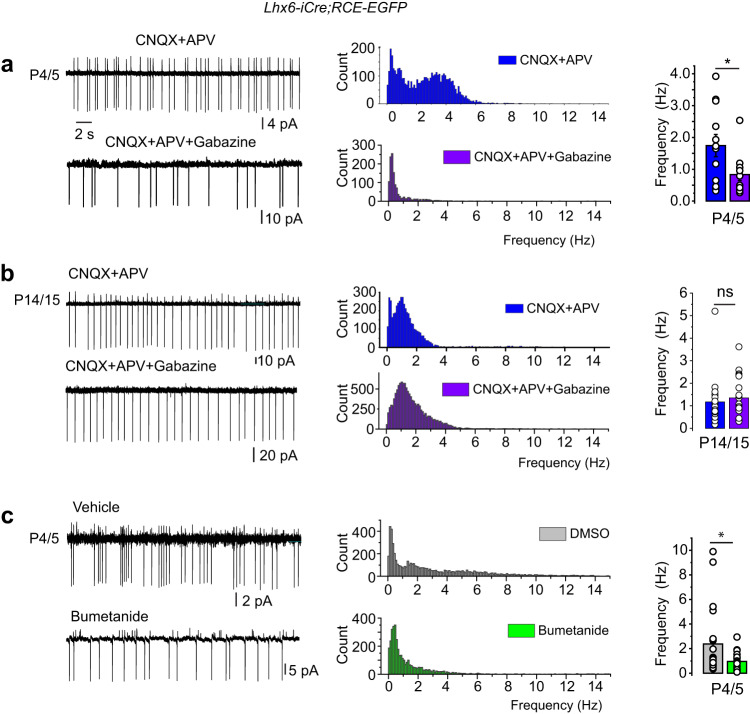
Effects of blockers of GABA_A_R synaptic transmission on CGINs ongoing activity before and after GABA polarity shift. **a**, **b** (left) Representative cell attached recordings of ongoing CGINs activity, in the presence of the ionotropic glutamatergic blockers CNQX (10 µM) + APV (40 µM), and the subsequent application of Gabazine (10 µM) at P4/5 (**a**) or P14/15 (**b**). **c** (left) Representative P4/5 cell- attached recordings of ongoing CGINs activity in vehicle solution (DMSO) or in the presence of bumetanide (10 µM) applied 40 min before and during the recordings. **a**–**c** (middle) Pooled instantaneous frequency distributions of CGINs spontaneous spikes at the ages and conditions of (**a**–**c**) left. **a**–**c** (right), Mean frequencies of ongoing activities in the presence of CNQX-APV (blue), of CNQX-APV-Gabazine (violet), DMSO (gray) and bumetanide (green), at the indicated ages. P4/5 CNQX-APV: *n* = 12 (*N* = 6); P4/5 CNQX-APV-Gabazine: *n* = 11 (*N* = 7); P14/15 CNQX-APV: *n* = 17 (*N* = 6); P14/15 CNQX-APV-Gabazine: *n* = 22 (*N* = 7); P4/5 DMSO: *n* = 21 (*N* = 8); P4/5 bumetanide: *n* = 20 (*N* = 7). All means ± s.e.m (Supplementary Table 9). *n* = number of cells, *N* = number of mice.

Spontaneous firing of CGINs was already present at P0 at a low mean frequency (2.3 ± 0.1 Hz, *n* = 768 events, 11 cells, *N* = 4 mice). Then, from P2/3 to P7/8, CGINs firing pattern was irregular with bursting periods as attested by the presence of frequencies up to 14 Hz and over (Supplementary Fig. 8). To evaluate the participation of spontaneous GABA_A_R synaptic activity to ongoing CGIN activity, we tested the effect of GABA_A_R synaptic blockers and recorded spontaneous GABA_A_R-mediated PSCs before and after GABA polarity shift (P10). Ionotropic glutamate receptor blockers were first applied to suppress afferent AMPA and NMDA receptor-mediated spontaneous activity. Gabazine was then applied in addition to APV and CNQX. It had a strong effect at P4/5 suppressing all frequencies above 4 Hz (bursts), and significantly decreasing the mean frequency of activity from 1.7 ± 0.3 Hz to 0.8 ± 0.2 Hz (*n* = 12, *N* = 6 mice and *n* = 11 cells, *N* = 7 mice, respectively) (Fig. 3a and Supplementary Table 9). In contrast, applied at P15 after GABA polarity shift, gabazine had not a significant effect (Fig. 3b). Bath application of bumetanide, a NKCC1 chloride importer antagonist, at P4/5, suppressed bursts and significantly decreased the mean firing frequency from 2.4 ± 0.6 Hz in vehicle (DMSO) to 1.0 ± 0.1 Hz (*n* = 21 cells, *N* = 8 mice and *n* = 20 cells, *N* = 7 mice, respectively; *p* = 0.03) (Fig. 3c and Supplementary Table 9).

In agreement with the above results, spontaneous outward postsynaptic spontaneous currents (PSCs, *V*_H_ = +10 mV) sensitive to picrotoxin, were present from E18 to P15. They had a bursty pattern in 20–30% of CGINs between P0 and P7 and their mean frequency dramatically increased before GABA polarity shift, from 0.76 ± 0.15 Hz at P4/5 to 2.84 ± 0.70 Hz at P6/7 (*n* = 10 cells, *N* = 8 mice and *n* = 9 cells, *N* = 4 mice respectively). These GABA_A_R-mediated PSCs had a mean amplitude of 43.1 ± 4.2 pA at E18 (*n* = 9 cells, *N* = 5 mice). They did not significantly vary thereafter up to P4/5 (Supplementary Figs. 9, 10, Supplementary Table 10). These results strongly suggest that GABA_A_R synapses afferent to CGINs are present and spontaneously active at birth. Interestingly, the abrupt and significant change in the frequency of GABA sPSCs between P4/5 and P6/7 coincides with the significant increase in the area under the Sholl curve and the critical radius at the same ages (see Fig. 1c, d), indicators of a significant increase in the dendritic tree surface.

### Shift of the cortico-striatal response from a long GABA_A_R-mediated excitation to a pause

**Fig. 4:**
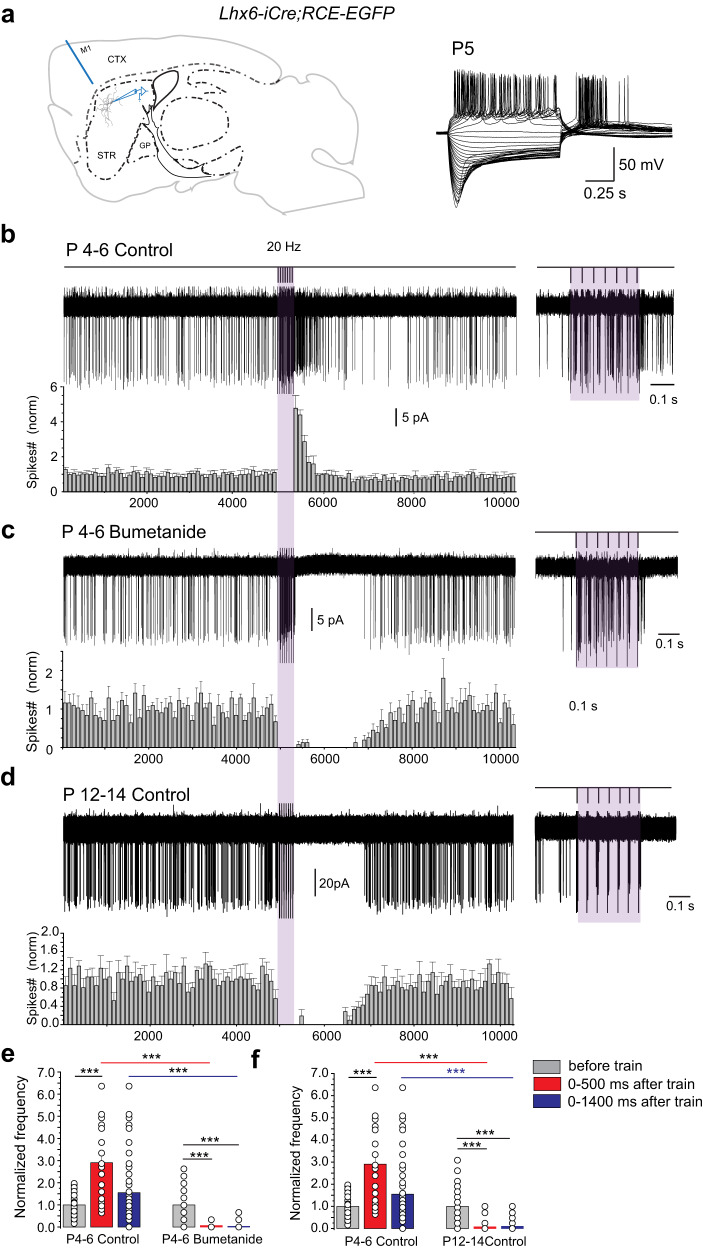
Developmental shift from excitatory to inhibitory (pause), of the CGIN post train GABAergic response to cortical stimulation. **a** (left) Schematic of a parasagittal slice showing the locations of the extracellular stimulating and patch clamp recording electrodes (CTX cortex; GP globus pallidus; M1 region of motor cortex; STR dorsal striatum) (drawing by C.H.). Right, Typical CGIN voltage responses to current pulses. **b** (top) Stimulation protocol (20 Hz, 7 stimuli); **b**–**d** (left), representative superimposed consecutive cell-attached responses (20–30 traces) to cortical train stimulation and mean frequency histograms; right: responses within the train at extended time scale to show the efficacy of the cortical stimulation. **b** Responses at P4–6 in ACSF (control, *n* = 8 cells (*N* = 4)), or (**c**), in the presence of bumetanide (10 µM) applied 40 min before and during the recordings (*n* = 6 cells (*N* = 3)). **d** Response at P12–14 in ACSF (control, *n* = 6 cells (*N* = 3)). **e**, **f** Mean number of spikes during two different time windows after train stimulation (0–500 ms, 0–1400 ms) normalized to spikes counts before train P4–6 control vs P4–6 bumetanide (**e**) or P4–6 control vs P12–14 control (**f**)). All means ± s.e.m (Supplementary Table 11). *n* = number of cells, *N* = number of mice.

GABA_A_R-induced depolarizations would impact the activity of cortico-striatal and intra-striatal cholinergic-GABAergic microcircuits only if these are functional at this stage. The cortically evoked pause response of cholinergic interneurons is considered an important element in cortico-striatal interactions and targeted motor behavior^27,28^. Knowing that cortical afferents are already present and functional from P0 (Supplementary Fig. 11 a, b)^29^ or P3/4^30^, we tested whether cortical stimulation generated a similar pause response in immature slices. At P4–6, in non-invasive, voltage-clamp, cell-attached recordings, cortical stimulation (7 stimuli at 20 Hz) evoked spikes during the stimulation period immediately followed by a transient increase of spiking frequency (post-train excitation) compared to ongoing base line frequency before the stimulation (460 ± 30%, duration ~500 ms, *n* = 8 cells, *N* = 4 mice) instead of the pause response recorded at P12–14 (duration ~1200 ms, *n* = 6 cells, *N* = 3 mice) (Fig. 4a, b, d, f, and Supplementary Table 11). To test whether the cortico-striatal connection was indeed excitatory at P4–6 because of elevated intracellular chloride level in immature CGINs, we performed the same experiment in the presence of bumetanide to lower intracellular chloride concentration. Bath application of bumetanide completely abolished the excitatory response and replaced it by a pause (duration ~1400 ms, *n* = 6 cells, *N* = 3mice, Fig. 4c, e) suggesting that GABA_A_- receptor-mediated post-train excitation was due to the high [Cl^−^]_i_ level present in CGINs at P4–6. Moreover, the P4–6 cortico-striatal excitatory response was totally supressed by bath application of picrotoxin (Supplementary Fig. 12, Supplementary Table 12).

These experiments strongly suggested that the cortico-CGINs microcircuitry including striatal GABAergic interneurons, are already functional during the first postnatal week. At P4–6 the classical pause response was absent and replaced by an excitatory response in keeping with the GABA_A_Rs-mediated excitation of CGINs generated by isoguvacine at P4–6 (see Fig. 2). The mature-like “pause-response” appeared later, at P12–14, once the excitatory-inhibitory shift of GABA polarity had occurred.

### Shift of the polysynaptic microcircuit connecting cholinergic interneurons from excitatory to inhibitory

**Fig. 5:**
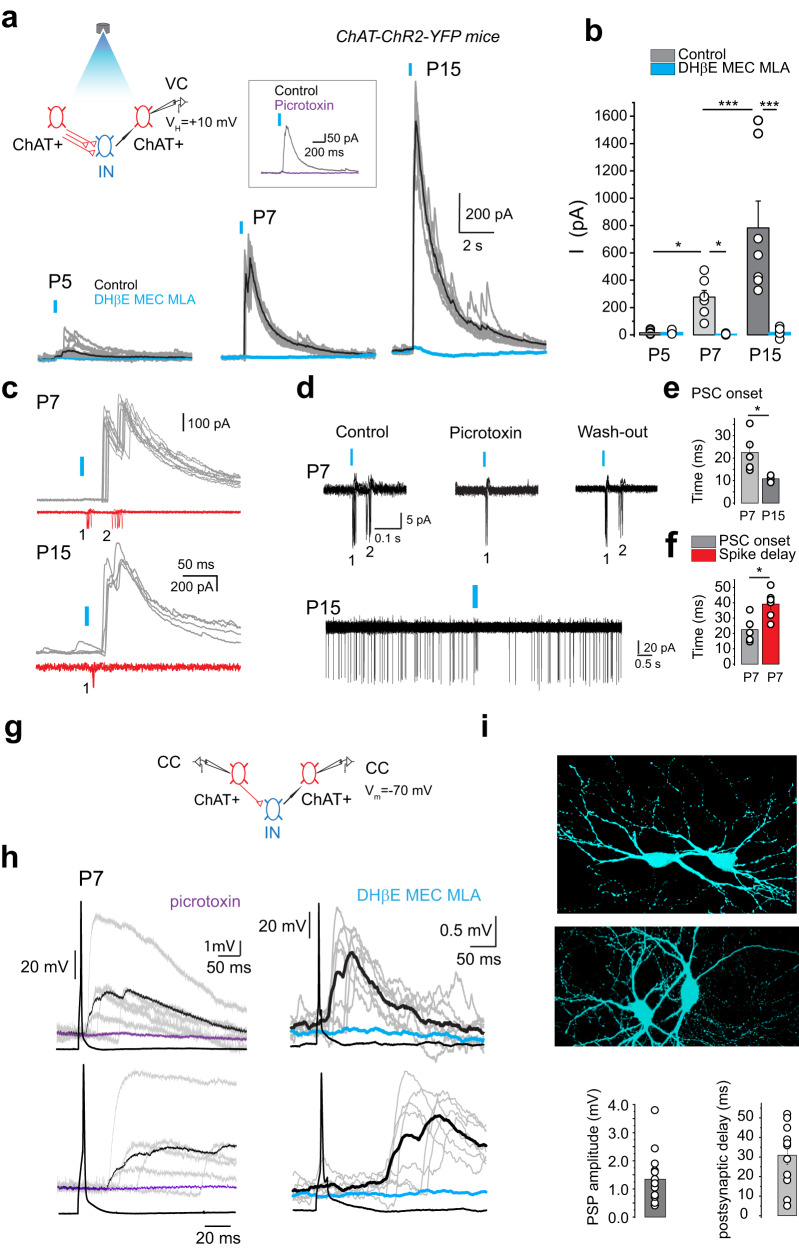
Developmental shift from excitatory to inhibitory of the polysynaptic response between cholinergic interneurons. **a** (top left), schematic representations of the ChAT^+^-ChAT^+^ polysynaptic microcircuit and experimental setups for a-c; IN: GABAergic interneurons. **a** (bottom), whole-cell recordings of superimposed polysynaptic PSCs evoked in ChAT^+^ interneurons in response to optogenetic stimulation of ChAT-Rho^+^ presynaptic axonal terminals (blue vertical bars) at the indicated ages (low [Cl^-^], Cs^+^-based intracellular solution, see Methods). Nicotinic receptor antagonists (MEC (10 µM), MLA (0.1 µM), and DHβE (10 µM)) blocked the polysynaptic PSCs (blue trace). **a** (inset), Block of the polysynaptic PSCs by picrotoxin (50 µM, P7). **b** Polysynaptic PSCs mean amplitudes P4/5 control: *n* = 10 (*N* = 6); P4/5 NA: *n* = 5 (*N* = 3); P7 control: *n* = 7 (*N* = 5); P7 NA: *n* = 6 (*N* = 3); P14/15 control: *n* = 7 (*N* = 4); P14/15 NA: *n* = 6 (*N* = 3). **c** Superimposed consecutive cell-attached recordings of spikes (red) and of the corresponding whole-cell polysynaptic PSCs (gray) evoked in the same ChAT^+^ interneuron in response to optogenetic stimulation of ChAT-Rho^+^ presynaptic axon terminals at the indicated ages. The short latency spikes (1) corresponded to the direct activation of the recorded cells by light. At P7, the delayed spikes (2) corresponded to the peak of the polysynaptic PSC (**c**, top) whereas at P15 this delayed response was absent (c, bottom). **d** (top), Superimposed consecutive cell-attached responses of a ChAT^+^ interneuron to optogenetic stimulation at P7. As in c, the delayed spikes (2) corresponded to the polysynaptic excitatory response. Picrotoxin (50 µM) reversibly blocked the delayed spikes (2) but not the early spikes (1). **d** (bottom), Superimposed consecutive cell-attached inhibitory polysynaptic response of a ChAT^+^ cell to the train of optogenetic stimuli at P15. **e** Delays of polysynaptic PSCs onsets at P7 and P15 (P7: *n* = 5 (*N* = 3); P14/15: *n* = 4 (*N* = 3)). **f** Latencies of polysynaptic PSCs onsets and delay between the first and 2nd spikes at P7 (*n* = 6 (*N* = 4)). **g** experimental set up for (**i**, **h**). **h (top traces)**, Paired whole-cell recordings (high [Cl^-^], K^+^-based intracellular solution, see Methods) of PSPs evoked in a postsynaptic ChAT^+^ neuron in response to a spike generated in the presynaptic ChAT^+^ neuron in control ACSF (gray traces and mean in black) and in the presence of picrotoxin (50 µM, violet) or nicotinic receptors antagonists (blue). Bottom traces show the same experiment at expended time scale and quantification of the amplitude and delay of the postsynaptic PSP in control conditions. **i** The biocytin-filled, polysynaptically-connected ChAT^+^ cells recorded in (**h**) (Scale bar 30 µm). CC: current clamp mode, VC: voltage clamp modes All means ± s.e.m (Supplementary Table 13). *n* = number of cells, *N* = number of mice.

The activity pattern of striatal cholinergic cells is shaped by a strong and widespread polysynaptic network^14^, mainly mediated by striatal tyrosine hydroxylase-expressing interneurons (THINs)^13^. To record how this polysynaptic microcircuit between cholinergic interneurons works in immature condition, we used ChAT-ChR2-EYFP mice to identify and light stimulate EYFP^+^ (ChAT^+^) presynaptic axon terminals (see methods and Supplementary Fig. 13). We recorded the evoked outward polysynaptic response from a single postsynaptic EYFP^+^ neuron in whole cell configuration (low chloride intracellular solution) and voltage-clamp mode (*V*_H_  =   + 10 mV). UV light stimulation evoked a tiny and rare response at P5 but generated an outward polysynaptic postsynaptic current (PSC) at P7 (mean amplitude 276 ± 49 pA, *n* = 7 cells, *N* = 5 mice) and at P15 (mean amplitude 783 ± 196 pA, *n* = 7 cells, *N* = 4 mice) (Fig. 5a, b and Supplementary Table 13). Bath application of the three nicotinic receptor antagonists, dihydro-β-erythroidine (DHβE, 10 µM) a competitive antagonist of α4β2 containing nAChRs, mecamylamine (MEC), a nonselective, noncompetitive antagonist of nAChRs, and methyllycaconitine (MLA), a selective antagonist of α7-containing nAChRs) or picrotoxin (50 µM) (Fig. 5a, b and inset) totally blocked the polysynaptic PSC suggesting that it is generated via the activation of nAChR-activated GABAergic interneurons by light-stimulated EYFP^+^ presynaptic axon terminals (Fig. 5a top left). It also suggests that the GABAergic polysynaptic microcircuits between ChAT^+^ interneurons begun to be detectable and functional between P5 and P7.

To test whether the polysynaptic ChAT^+^-ChAT^+^ connection via GABAergic interneurons are excitatory before GABA polarity shift (P10), we performed at P7 the same optogenetic stimulation of EYFP^+^ presynaptic axon terminals recording the response of postsynaptic EYFP^+^ cells in cell-attached, voltage-clamp mode to leave [Cl^-^]_i_ intact (Fig. 5c, red traces). This is followed by whole-cell recordings of GABAergic PSCs in the same cell (Fig. 5c, gray traces). Optogenetic stimulation evoked in EYFP^+^ cells a first spike (1) with a short latency (due to direct optogenetic stimulation of the YFP^+^ recorded cell) and delayed 1-2 spikes (2) at the peak of the GABAergic PSC (mean delay between 1st and 2nd spikes = 39.1 ± 3.7 ms, *n* = 6 cells, *N* = 4 mice) (Supplementary Table 13). At P15 the same stimulation evoked only the first spike (1) (Fig. 5c, bottom). Delayed spikes (2) in cell-attached mode appeared after the onset of the GABAergic PSC (PSCs onset at P7 = 22.5 ± 3.8 ms, *n* = 5 cells, *N* = 3 mice) (Fig. 5c top, e, f). Picrotoxin totally abolished the delayed spikes (2) (Fig. 5d, top), suggesting that, at P7, the response is mediated by excitatory GABAergic interneurons participating in the polysynaptic connections between cholinergic interneurons. In contrast, optogenetic train at P15 evoked an inhibition of CGINs, as already described^13,14^ (Fig. 5d, bottom).

To test whether the polysynaptic ChAT^+^-ChAT^+^ microcircuit operates directly via GABAergic interneurons and to exclude light-stimulated extrastriatal cholinergic inputs^31^, we performed dual whole-cell current-clamp recordings from pairs of EYFP^+^ cells (Fig. 5g, h). At P7, a single action potential evoked in the presynaptic EYFP^+^ cell elicited a post-synaptic potential (PSP) in a postsynaptic EYFP^+^ cell in 13% of the tested pairs (15 out of 116), with an averaged amplitude of 1.35 ± 0.23 mV. This PSP was completely blocked by picrotoxin (*n* = 3 cells, *N* = 3 mice) or nicotinic antagonists (*n* = 4 cells, *N* = 4 mice) suggesting that a single action potential in a presynaptic cholinergic interneuron can evoke depolarizing GABAergic potentials in other cholinergic interneurons via nAChR-activated GABAergic interneurons (Fig. 5h, i). A monosynaptic cholinergic/GABAergic connection between CGINs could explain the single spike-evoked PSP recorded in Fig. 5h, but this seems unlikely since this PSP is completely abolished by picrotoxin (in the absence of nAChR antagonists) and its delay (30.9 ± 4.9 ms) is too long for a monosynaptic response.

In ChR2-ChAT-EYFP striatal slices, CGINs could not be differentiated during the recording session. To confirm that CGINs are involved in polysynaptic nicotinic receptor-mediated networks, we performed synchronous activation of CGINs by cortical stimulation in slices from Lhx6-EGFP mice. Cortically evoked, picrotoxin-sensitive, PSCs recorded in CGINs were significantly inhibited by application of nicotinic antagonists (by 23 ± 4% at P7, *n* = 9 cells; *N* = 3 mice and by 58 ± 7% at P15, *n* = 9 cells, *N* = 3 mice). Interestingly, this inhibition of CGINs activity was significantly stronger at P14 than at P7 (Supplementary Fig. 14a–d and Supplementary Table 14). This suggested that a strengthening of the polysynaptic GABAergic network between CGINs takes place after P7 (see Fig. 5a, b and Supplementary Fig. 14e).

### Early GABA_A_R-mediated excitatory drive and GABA polarity shift

**Fig. 6:**
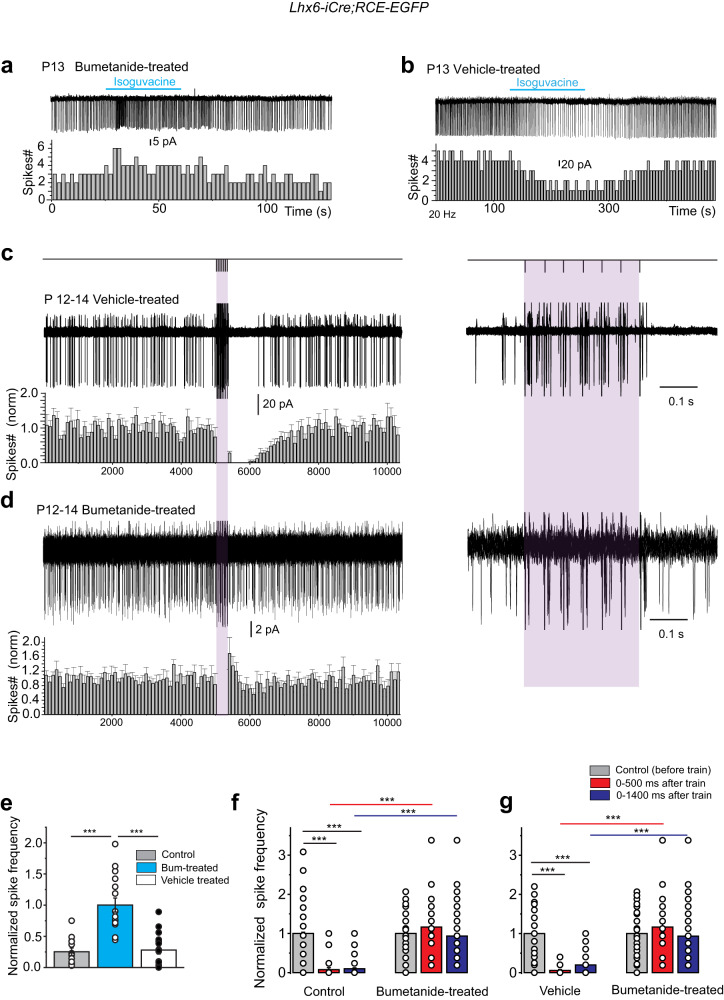
Absence of developmental shift of the GABA_A_R-mediated response and of cortico-striatal GABAergic pause response after early chronic treatment of pups with the NKCC1 blocker bumetanide. **a**, **b** Representative cell-attached recordings, and corresponding mean frequency histograms of the effect of isoguvacine (10 μM) on a P13 CGIN ongoing activity in slices from bumetanide (**a**) or vehicle (**b**) treated pups (see methods). **c** (top) Experimental protocol: cortical stimulation (20 Hz, 7 stimuli); **c**. **d** (left), representative cell-attached, superimposed (*n* = 15), consecutive responses to cortical train stimulation of P12- P14 CGIN in slices from bumetanide-treated mice (**d**) and vehicle (**c**) and mean frequency histograms. Note the absence of the pause response (**d**)**. c**, **d** (right), Responses within the train at extended time scale to show the efficacy of cortical stimulation. **e** Quantification of data shown in (**a**, **b)** (bumetanide-treated: *n* = 16 cells (*N* = 3); vehicle-treated: *n* = 17 cells (*N* = 3). **f**, **g** Quantification of data shown in (**c**, **d)**: mean number of spikes during two different time windows after cortical train stimulation (0–500 ms, 0–1400 ms) normalized to spikes counts before the train (bumetanide-treated: *n* = 7 cells (*N* = 3); vehicle-treated: *n* = 10 cells (*N* = 3)). All means ± s.e.m (Supplementary Table 15). *n* = number of cells, *N* = number of mice.

To determine the possible role of the transient excitatory phase of cholinergic microcircuits that takes place up to the end of the first postnatal week, we treated pups with i.p. bumetanide between P4 and P8 i.e., before GABA polarity shift (P10, see methods). We then performed whole cell current clamp recordings of CGINs activity in striatal slices from Lhx6-EGFP mice aged P13 previously treated with bumetanide. In slices from bumetanide-treated mice, isoguvacine application at P13 (*n* = 16 cells, *N* = 3 mice) no longer inhibited CGINs spontaneous activity (Fig. 6a, e) in contrast to slices from vehicle treated mice (*n* = 17 cells, *N* = 3 mice) (Fig. 6b, e). In bumetanide-treated mice, at P12–14 the pause-response evoked in CGINs by cortical stimulation (*n* = 7 cells) disappeared (*n* = 7 cells, *N* = 3 mice, Fig. 6d) in contrast to the presence of the pause response in striatal slices from control pups (*n* = 6 cells, *N* = 3 mice, Figs. 4d, f, 6f) and striatal slices from vehicle-treated pups (p12–14, *n* = 10 cells, *N* = 3 mice, Fig. 6c, g) (Supplementary Table 15). These results point out a crucial role of the transient excitations of striatal polysynaptic microcircuits during the first postnatal week in the developmental shift of GABA polarity in CGINs and control functioning of CGINs-GABAergic microcircuits during the second postnatal week.

## Discussion

**Fig. 7:**
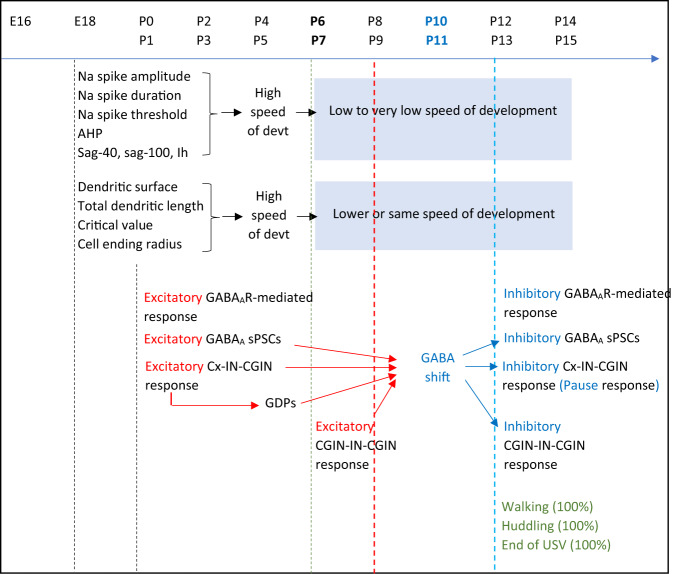
Time course of the different parameters studied during the development of the dorsal striatum from E18 to P15. The intrinsic morphological and electrophysiological parameters of striatal cholinergic interneurons develop rapidly during the first postnatal week, before the GABA polarity shift. Simultaneously, the excitatory command evoked by spontaneous GABA_A_R-evoked PSPs in the striatal network favors the occurrence of GABA polarity shift during the second postnatal week. Then the striatal cholinergic networks behave as in the adult. Interestingly, walking, huddling and the end of USV calls occur in all pups tested after GABA polarity shift.

At the ages from the E16 to P15, before full acquisition of quadruped locomotion, the development of mice CGINs and CGINs microcircuits of the dorsal striatum follows the sequence summarized in Fig. 7. CGINs intrinsic parameters and synaptic connections mature rapidly during the first postnatal week, thus generating an excitatory nicotinic drive. Concomitantly and before GABA polarity shift (P10), cholinergic-GABAergic microcircuits are excitatory instead of inhibitory, the cortico-striatal “pause” response is excitatory and cholinergic interneurons are interconnected by a polysynaptic excitatory network. By the beginning of the second week, when the GABA_A_R-mediated response becomes inhibitory, the cortico–striatal response shifts to the classical pause response and cholinergic interneurons become connected by a polysynaptic inhibitory network. Interestingly, the adult-like functioning of CGINs microcircuits is concomitant with locomotor and exploratory behavior^32^. Imposing an early GABA polarity shift by chronic parenteral administration of bumetanide to mouse pups, prevents the subsequent GABA polarity shift in CGINs and the generation of the cortico-striatal pause response. This illustrates the importance of this critical period and its long-lasting effects. It also suggests that transient excitations in striatal microcircuits due to the spontaneous activation of excitatory GABAergic interneurons via their afferent ionotropic glutamatergic, cholinergic (nicotinic) and GABAergic (GABA_A_) synapses, are instrumental in generating GABA polarity shift.

If CGINs had their final number of primary dendrites already at E16, the maturation of their dendritic tree mainly occurred during the first postnatal week. From E16 to P7, their overall dendritic length and dendritic surface increased by a factor of 3 and the number of crossing intersections more than doubled. A similar speed of maturation was observed for intrinsic electrophysiological parameters. Action potentials became both larger (amplitude was multiplied by 3) and faster (half duration was divided by 5) and their spontaneous bursting pattern peaked during the first postnatal week. This suggests that many intrinsic currents develop rapidly during the first postnatal week including the voltage-gated Na^+^ and delayed rectifying K^+^ currents responsible for spike amplitude and duration, the voltage-gated Ca^2+^ and Ca^2+^-dependent K^+^ currents responsible for bursts and AHPs, and the subthreshold cationic H current responsible for sag, pacemaker and rebound spikes. After P7, the speed of development significantly slowed down. As in adult striatum^33,34^, early cholinergic interneurons pacemaker activity results from the hyperpolarization-activated current I_h_, which repolarizes the membrane between spikes, activates the persistent sodium current I_NaP_, mainly present from the second postnatal week^20^ and drives membrane potential to action potential threshold.

Due to the early excitatory effect of GABA_A_ receptor-mediated currents and the early functionality of GABAergic and glutamatergic synapses afferent to CGINs, spontaneous currents mediated by ionotropic GABA_A_ and glutamate receptors both increase spontaneous CGINs activity throughout the first postnatal week, before GABA polarity shift. Spontaneous GABA_A_R-mediated PSCs were already present at E16 and at P5 the blockade of GABA_A_R (after the blockade of ionotropic glutamate receptors) strongly decreased frequency of CGINs activity. In the adult cholinergic interneurons, GABAergic synapses represent 60% of their afferents^25,35^. These include GABAergic synapses from local feedforward somatostatin positive, low threshold spike (PLTS), tyrosine hydroxylase positive (THIN), neuropeptide Y-expressing neurogliaform (NGF NPY), and fast adapting (FAI) GABAergic interneurons^8,10,13,36–38^, axon collaterals of spiny neurons^39^, extrinsic arkypallidal neurons of the globus pallidus^40^ and cortical GABAergic projection neurons^41^. These afferences, or at least some of them, are spontaneously active during the first postnatal week.

Cortico-striatal glutamatergic connections were already functional at P0^29^ or P3/4^30^. By directly activating CGINs and GABAergic interneurons, they evoked a long-lasting picrotoxin, and bumetanide-sensitive excitation of CGINs instead of the classical pause response recorded at the end of the second postnatal week and later^4^. Several observations suggest that CGINs early activity is probably driven by immature cortical activities. Developing cortical pyramidal neurons first generate calcium plateaus, followed by cortical early network oscillations (cENOs) synapse-driven by glutamatergic transmission around P2-P3, and GDPs at P6-P8^42,43^. The early pattern of CGINs activity in fact also involved burst of Na^+^ spikes, most likely generated by calcium plateaus around P2–4 (synchronized by gap junctions and supported by the activation of voltage-gated intrinsic conductance), followed at P4-P7 by shorter bursts of activity probably resulting from giant depolarizing potentials (GDPs), together with single Na^+^ spikes generated by the previously described cascade of subthreshold currents. Cortically driven GDPs have been previously recorded in developing striatal spiny projection neurons^29^. Afferent cortical excitations may be fueled during the first postnatal week by sensory information from reflexes and early pivoting and crawling movements of pups^44^ recorded in the present study before GABA polarity shift.

The polysynaptic network between cholinergic interneurons was functional and excitatory around P7, providing a supplementary excitatory surge activating many cholinergic interneurons before GABA polarity shift. This stands in contrast with the adult situation where this polysynaptic network is inhibitory, as cholinergic interneurons recruit local THINs via nAChRs activation and drive synchronized GABAergic pauses in multiple neighboring cholinergic interneurons^13,14^. Altogether, the fast maturation of CGINs, their early connections to excitatory glutamatergic and GABAergic afferents, their low spike threshold, spontaneous bursting pattern, and participation in recurrent excitatory polysynaptic GABAergic circuits likely underlines the functional role they play in the development of striatal networks.

GABA polarity shift occurred in CGINs during the second postnatal week, between P8 and P12, as observed in other neuronal types in a large varieties of brain structures and the underlying mechanisms have been extensively investigated^21^. During early postnatal development (until P8–10), GABA exerts a depolarizing and excitatory action as immature neurons accumulate intracellular Cl^-^ ions via the cotransporter NKCC1. Then, GABA_A_R-mediated depolarizations remove the Mg^2+^ block from NMDA receptors, activate voltage-gated Ca^2+^ currents, increase the intracellular Ca^2+^ levels^45,46^ and trigger intracellular signaling cascades, leading to an upregulation of the chloride exporter K^+^−2Cl^-^ (KCC2)^47^. KCC2 becomes predominantly expressed, lowers intracellular Cl^-^ concentration which converts GABAergic transmission to inhibitory^48,49^.

Chronic bumetanide treatment of pups, from P4 to P8, prevented the GABA inhibitory shift and the development of the cortico-striatal pause response at P12-P14 in CGINs. The absence of inhibitory GABA_A_-receptor mediated response at P12–14 suggests that bumetanide-sensitive excitatory activity in local cortico-striatal and striatal microcircuits present throughout the first postnatal week (see Figs. 2, 4, 6 and Supplementary Fig. 12), is a critical period underlying the change of GABA polarity in CGINs. In neuronal hippocampal cultures, calcium influx into neurons supported by depolarizing GABA_A_ transmission is instrumental for GABA polarity switch, since it is prevented by GABA_A_Rs blockers from Day 2 to Day 14 in vitro^48^. Similar long-lasting effects of chronic bumetanide treatment or blockade of GABA_A_R-mediated transmission, or premature KCC2 expression have been observed in rat pups or tadpoles. Preventing GABA polarity shift during a critical pre-postnatal period lead to the lengthening of the time window for visual cortex plasticity^50^, the disruption of the balance between excitatory and inhibitory inputs in cortical pyramidal neurons^51^ and retinotectal neurons^52^ or the disruption of long term plasticities in CA1 hippocampal neurons^53^. Collectively, these observations illustrate the importance of the timing of the GABA polarity shift. The bumetanide treatment, which was not specifically targeted to the striatum, affected all brain GABAergic synapses including the numerous GABAergic afferents to striatal cholinergic interneurons^1,39^. Future experiments, using notably chemogenetic tools, will more precisely determine whether the excitation of cholinergic interneurons by GABAergic interneurons during the first postnatal week plays a determining role in the change of GABA polarity in the dorsal striatum.

Because of the early excitatory GABA_A_R-mediated activities and the early ongoing excitatory cholinergic drive, cholinergic-GABAergic microcircuits of the striatum play a significant early role in the development of striatal networks, before playing their well-known role in motor activities. Since CGINs are also present in the ventral striatum and the cholinergic-GABAergic microcircuits similar^25^, the present results could apply to developing microcircuits of the ventral striatum before this structure plays a role in reward-based decisions that usually accompany motor activities.

## Methods

### Animals

We generated transgenic mice Lhx6-iCre^+/−^;RCE-EGFP^+/−^ by crossing Lhx6-iCre^+/−^;RCE-EGFP^+/−^ mice (generous gift from Prof. Gordon J. Fishell) with wild-type swiss mice (CE Janvier, France). EGFP expression (which indicates present or past expression of Lhx6) as well as electrophysiological signature and ChAT-positive posthoc immunohistochemistry were used as markers of CGINs (ChAT^+^ EGFP^+^ interneurons). For optogenetic experiments, we crossed hemizygous ChAT-ChR2-EYFP mice (6.Cg-Tg(Chat-COP4*H134R/EYFP,Slc18a3)6Gfng/J, The Jackson Laboratory) on a *C57BL*/*6J* genetic background with swiss mice (CE Janvier, France) to re-direct them on a swiss genetic background. Matings were done overnight by placing one male with two females. Vaginal plugs were checked early the following morning at 7AM and noted as E0.5 day of gestation. Mice were maintained on a 12-h light cycle (7AM–7PM) with ad libitum access to food and water. Experiments were performed in both males and females, in agreement with the European community council directives (2010/63/UE). Protocols were approved by the local French ethical committee for animal experimentation (#19196) and validated by the French Ministry of Higher Education, Research and Innovation (authorization #19196-2018071214167976).

### Time window of the study (E16-P15)

The starting date of our study was the embryonic day E16 since it was the earliest date for optimal reliability of ChAT immunostaining. To study the early putative role of cholinergic interneurons microcircuits before they got involved in learning behaviors and habits, we decided that the ending date of our study should corresponded to the date of full acquisition of quadruped ambulation. We studied this parameter in two different contexts, in open field and during huddling in P2 to P15 Lhx6- EGFP mice (see Supplementary Methods). 100% of the pups were crawling and moving in an open field around P8, but they did not all properly walk with their belly off the floor before P14 (Supplementary Fig. 1a). Walking during huddling gave similar results. The different parameters of clusters and cluster switches stabilized between P12 and P15 (Supplementary Fig. 1c, d). Ultrasonic vocalization (USV) i.e., distress calls, completely stopped at P15 (Supplementary Fig. 1b), confirming pups’ ability to easily move towards sibblings/mother at that stage. For comparison, neurodevelopmental motor reflexes, such as surface righting, negative geotaxis, and cliff avoidance were acquired for all pups at P8, a week earlier than walking, (Supplementary Fig. 2 and methods). We therefore ended our study at P15.

### Chronic bumetanide treatment

Lhx6-iCre^+/−^; RCE-EGFP^+/−^ pups, received intraperitoneal (i.p.) injections of bumetanide (0.2 mg/kg, Sigma) or vehicle (2% dimethylsulfoxide (DMSO)) at 12-h intervals (twice daily at 7AM and 7PM) by the same experimenter from P4 to P8. Bumetanide or vehicle was diluted in physiological saline immediately before the injection to the final concentration of 0.2 mg/10 ml. The injected volume ranged from 20 µl to 60 µl per pup.

### Electrophysiology

Slice preparation, patch clamp recordings, extracellular and optogenetic stimulations were performed as previously described (ref. ^4^ and Supplementary Methods). Cell-attached recordings were performed 5–7 min after establishment of the gigaohm seal and after stabilization of base line. Patch pipettes were filled with extracellular solution. For whole-cell recordings, patch pipettes (World Precision Instruments, Sarasota, USA) were filled with either the “low” chloride intracellular solution (in mM): 130 K-gluconate, 10 Na-gluconate, 7 NaCl, 4 MgATP, 4 Na_2_-phosphocreatine, 10 HEPES and 0.3 GTP (pH 7.3 with KOH, 280 mOsmL^−1^) or the ”high” chloride intracellular solution (in mM): 105 K-gluconate, 30 KCl, 4 MgATP, 10 Na_2_-phosphocreatine, 10 HEPES and 0.3 GTP (pH 7.3 with KOH, 280 mOsmL^−1^). For PSCs recordings (evoked by electrical stimulation or light) a Cs-based low -chloride solution was used: 130 Cs-gluconate, 10 Na-gluconate, 7 NaCl, 4 MgATP, 4 Na_2_-phosphocreatine, 10 HEPES and 0.3 GTP (pH 7.3 with CsOH, 280 mOsmL^−1^). Biocytin (final concentration of 0.3–0.5%) was added to pipette solutions to label recorded neurons. Equilibrium potentials of chloride ions were −75 mV for the “low” and −37 mV for the “high” chloride K^+^-based solutions (25 °C). We discarded cells with leakage current over 40–50 pA. Whole-cell patch-clamp recordings were performed with EPC-9 amplifier and Patch Master software v2x73.2 (HEKA Elektronik, Germany) and filtered at 3–10 kHz. For cell-attached recordings, pipettes were filled with extracellular solution.

To determine current–voltage (*I*–*V*) relationships and calculate input resistance of cells, we recorded voltage responses (current-clamp mode) to a series of 1 s current pulses. For the hyperpolarization-activated SAG, we measured the ratio between the peak amplitude and the amplitudes at the end of the voltage response. For action potentials (AP) parameters, duration (half width) was measured at half of the maximal spike amplitude. After-hyperpolarization (AHP) was measured at its peak. To measure I_h_, we applied a series of 2-sec-long hyperpolarizing steps from −60 to −120 mV (voltage-clamp mode, increments: 5 mV, *V*_H_ = −60 mV). I_h_ amplitude was calculated as the difference between sustained and instantaneous currents. The exponential fit of initial (time-dependent) part of I_h_ was extrapolated back to the onset of the pulse to obtain instantaneous current value. The final value of the current at the pulse offset was used to estimate the steady-state current. I_h_ density was calculated as I_h_ (at −120 mV)/*C*_m_ where *C*_m_ is the cell capacitance. Clampfit 10.6.2.2 (Molecular Devices, LLC, USA) Igor 6.3.7.2 (WaveMetrics, Oregon, USA), OriginPro software were used for analysis.

### Extracellular and optogenetic stimulations

To electrically stimulate the cortico-striatal pathway, a bipolar Ni–Cr electrode was positioned in M1 motor cortex, just above corpus callosum (Fig. 4a). A train (7 stimuli, 20 Hz) of current pulses (25–50 μA, 100 µs) were delivered through the constant current bipolar stimulus isolator A365 (World Precision Instruments).

We activated the light-sensitive cation channel, channelrhodopsin-2 (ChR2) expressed by cholinergic interneurons, using a micro-mirror array system (Digital Micro-mirror Device, or DMD, Mosaic, Andor Technology, UK), and a high-power light-emitting diode (490 nm LED, CoolLED pE-4000, CoolLED Ltd., UK). We used PatchMaster and Andor iQ3 softwares to synchronize light stimulation and electrophysiological recordings. A brief light pulse (2–10 ms) reliably evoked spikes in cholinergic interneurons (Supplementary Fig. 13).

### Immunohistochemistry, reconstruction of biocytin-filled neurons and Sholl analysis

See^4^ and supplementary methods. We considered for analysis identified CGINs and cholinergic interneurons recorded from the dorsal striatum only.

### Drugs

SR 95531 hydrobromide (gabazine 5 μM, Tocris Bioscience, UK, Ref. 1262),2,3-dihydroxy-6-nitro-7-sulfamoyl-benzo[f]quinoxaline-2,3-dione (NBQX, 10 µM, NIH generous gift), isoguvacine (10 μM, Sigma-Aldrich, Ref. G002), DL −2-Amino-5-phosphonovaleric acid (APV, 40 µM, Sigma-Aldrich, Ref. A5282), TTX (10 nM, 1 µM, Abcam, Bristol, UK, Ref. 120055), picrotoxin (50 µM, Tocris Bioscience, UK, Ref. 1128), ZD7288 (100 µM, Tocris Bioscience, UK, Ref. 1000), bumetanide (10 μM, Sigma-Aldrich, Ref. B3023) and the cocktail of nicotinic receptor antagonists including mecamylamine hydrochloride (MEC, 10 μM, Tocris Bioscience, Ref. 2843/10), methyllycaconitine citrate (MLA, 0.1 μM, Tocris Bioscience, Ref. 1029/5) and Dihydro-β-erythroidine hydrobromide (DHβE, 10 μM, Tocris Bioscience, Ref. 2349/10) were directly added to the perfusion solutions.

### Statistics and reproducibility

Data were obtained in n cells from N mice as indicated in the text and figure legends. Note that the number of mice for in vitro experiments was equal to the number of litters and was always ≥3. Data from morphological experiments were computed in Prism 8 (GraphPad Software Inc, USA). They were tested for normality and homoscedasticity using the Shapiro–Wilk and Leven’s tests before performing statistical analysis. Comparisons between each age were done with the Kruskal–Wallis test followed by a Dunn’s multiple comparison test. Data from the electrophysiological and behavioral studies were analyzed with two-tailed-*t*-test or one-way -ANOVA -Fisher’s-LSD post-hoc tests. For morphological metrics we used one-way -ANOVA-Kruskal–Wallis- tests followed by Dunn’s-multiple-comparison post-hoc tests. Analyses were performed with Prism 8 (GraphPad Software Inc., USA) or OriginPro 019 (64-bit) 9.6.0.172 (OriginLab, USA). All data are presented as means ± s.e.m. *P < 0.05; **P < 0.01; ***P < 0.001. All data are computed in Supplementary Tables 1–15.

## Supplementary information

Supplementary Information

Description of Supplementary Data

Supplementary Data

Repoting Summary

## Data Availability

The numerical source data for the graphs are available in a Supplementary Data file with source data for Figs. 1–6 (grouped by experiment).
