# Supplementary material for: The early excitatory action of striatal cholinergic-GABAergic microcircuits conditions the subsequent GABA inhibitory shift
Source: Commun Biol. 2023 Jul 14;6:723. doi: 10.1038/s42003-023-05068-7 (PMC10349145; doi:10.1038/s42003-023-05068-7)
Supplement: Supplementary file 1 — Supplementary Information [file 42003_2023_5068_MOESM1_ESM.pdf]

Supplementary Methods and materials

SUPPLEMENTARY FIGURES

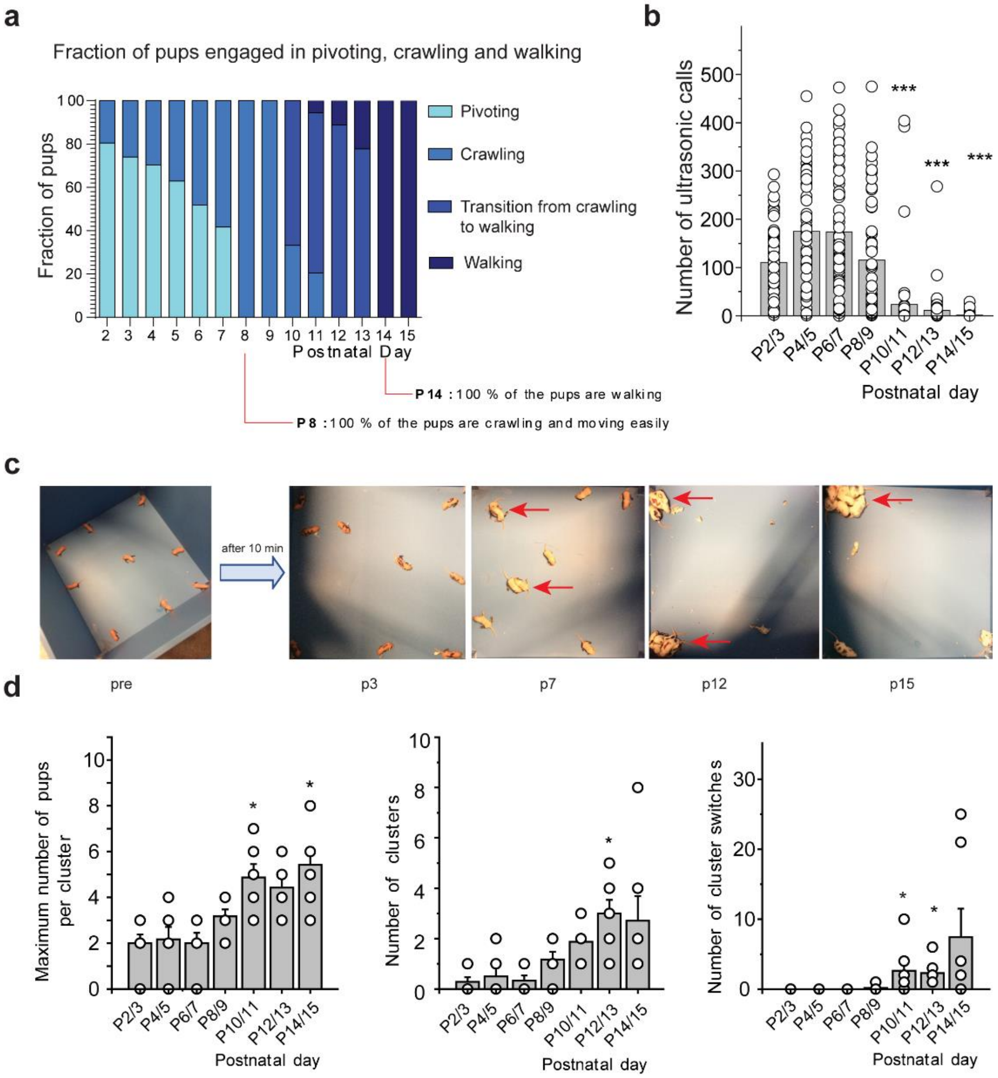

Supplementary Fig. 1. Identification of the time window between birth and walking acquisition
The behaviors of the same pups were studied from P2 to P15 in **a**, **b** or **c, d**. **a,** Mean percent of pups that acquired pivoting, crawling or walking in the open field at the indicated ages. (P2: N=18 (2 litters); P3: N=18 (2 litters); P4: N=18 (2 litters); P5: N=18 (2 litters); P6: N=27 (3 litters); P7: N=18 (2 litters); P8: N=27 (3 litters); P9: N=18 (2 litters); P10: N=27 (3 litters); P11: N=18 (2 litters); P12: N=27 (3 litters); P13: N=36 (4 litters); P14: N=27 (3 litters); P15: N=36 (4 litters). **b,** Number of calls (isolation-induced ultrasonic vocalizations) as a function of age (P2/3: N= 63 (7 litters); P4/5: N= 63 (7 litters); P6/7: N= 63 (7 litters); P8/9: N= 54 (6 litters); P10/11: N= 54 (6 litters); P12/13: N= 62 (7 litters); P14/15: N= 61 (7 litters). **c,** Representative image of the position of pups before huddling starts (left) and representative images of huddling behavior of pups when experimental sessions ended, at the indicated ages. Red arrowheads indicate individual clusters. **d**, Quantification of the average litter values ± SEM for three parameters as a function of age (see methods for the quantification of huddling behavior) (P2/3: N= 63 (7 litters); P4/5: N= 54 (6 litters); P6/7: N= 54 (6 litters); P8/9: N= 54 (6 litters); P10/11: N= 72 (8 litters); P12/13: N= 63 (7 litters); P14/15: N= 63 (7 litters). Supplementary Table 1. N=number of mice.

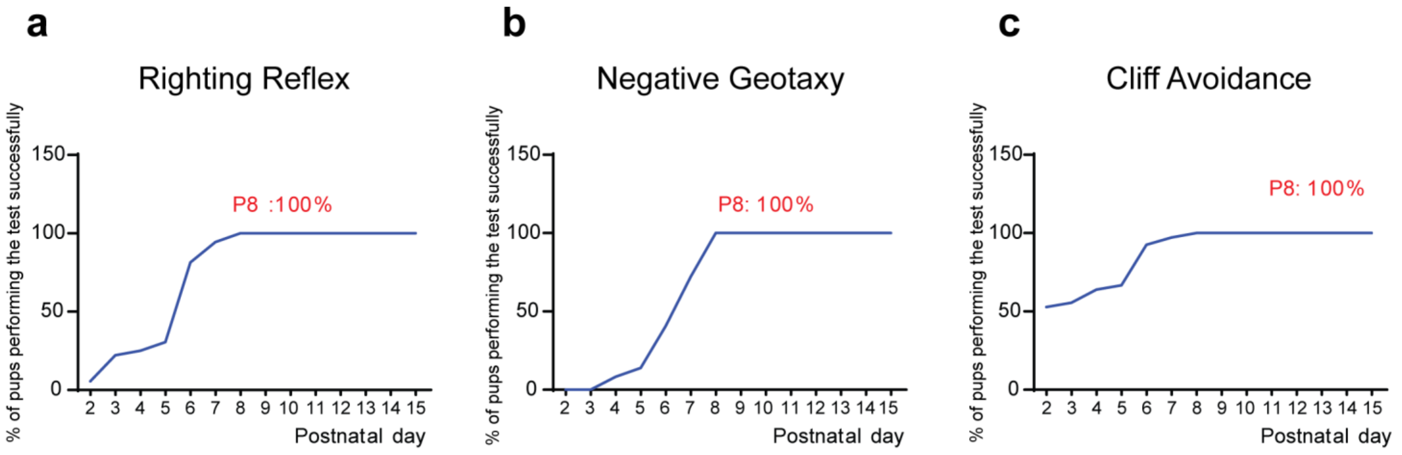

Supplementary Fig 2: time course of acquisition of three developmental motor reflexes: **a,** Righting Reflex (P2: N=36 (4 litters); P3: N=27 (3 litters); P4: N=36 (4 litters); P5: N=36 (4 litters); P6: N=27 (3 litters); P7: N=36 (4 litters); P8: N=27 (3 litters); P9: N=27 (3 litters); P10: N=27 (3 litters); P11: N=36 (4 litters); P12: N=27 (3 litters); P13: N=36 (4 litters); P14: N=27 (3 litters); P15: N=36 (4 litters); **b**, Negative Geotaxy (P2: N=36 (4 litters); P3: N=27 (3 litters); P4: N=36 (4 litters); P5: N=36 (4 litters); P6: N=27 (3 litters); P7: N=36 (4 litters); P8: N=27 (3 litters); P9: N=27 (3 litters); P10: N=27 (3 litters); P11: N=36 (4 litters); P12: N=27 (3 litters); P13: N=36 (4 litters); P14: N=27 (3 litters); P15: N=36 (4 litters); **c,** Cliff Avoidance (P2: N=36 (4 litters); P3: N=27 (3 litters); P4: N=36 (4 litters); P5: N=36 (4 litters); P6: N=27 (3 litters); P7: N=36 (4 litters); P8: N=27 (3 litters); P9: N=27 (3 litters); P10: N=27 (3 litters); P11: N=36 (4 litters); P12: N=27 (3 litters); P13: N=36 (4 litters); P14: N=27 (3 litters); P15: N=36 (4 litters). N=number of mice.

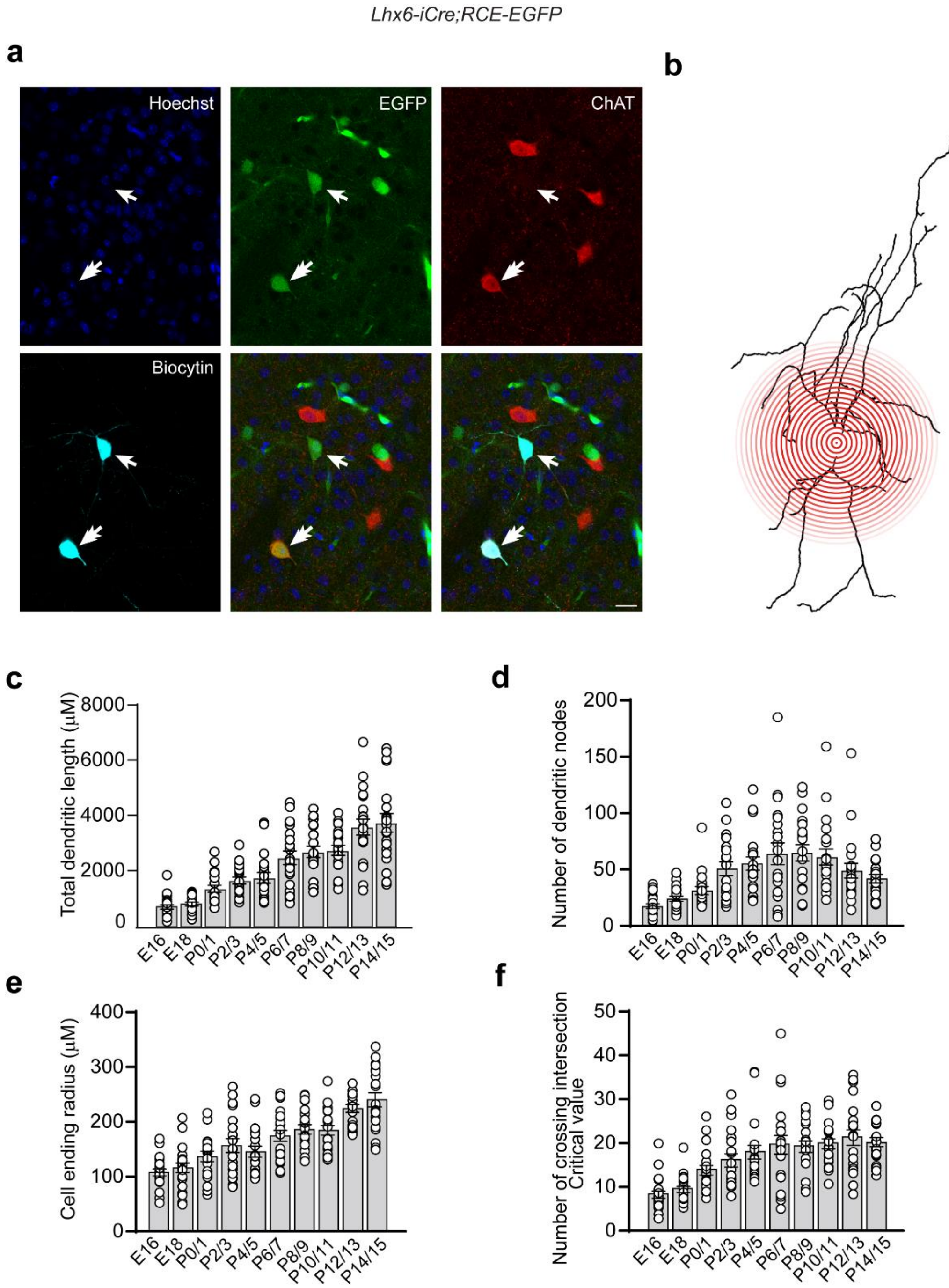

Supplementary Fig. 3: development of CGINs morphological properties (E16-P15).

a (top), Example of ChAT staining (red) validation in some EGFP+ (green) cells (Hoechst staining, cyan) in striatal slices from Lhx6-iCre;RCE-EGFP+ mice. The double arrow shows a EGFP+/ChAT+ cell, so called CGIN. The arrow shows a EGFP+/ChAT- cell. **a (bottom)**, same experiment but for biocytin-filled neurons. The double arrow shows a EGFP+/ChAT+ biocytin-filled CGIN. The arrow shows a EGFP+/ChAT-/biocytin-filled neuron. Scale bar: 20 µm. **b**. Illustration of Sholl analysis of a EGFP+/ChAT+/biocytin-filled, 3D reconstructed neuron. **c**, Total dendritic length. **d**, Mean number of nodes. **e**, Mean ending radius. **f**, Mean critical value (mean number of crossing intersections in Sholl analysis), of the dendritic trees of identified CGINs at the indicated ages. (E16: n=21, N=5; E18: n= 21, N=3; P0/1: n=20, N=6; P2/3: n=20, N=7; P4/5: n= 20, N=13; P6/7: n= 21, N=4; P8/9: n= 20, N=5; P10/11: n=21, N=12; P12/13: n=21, N=5; P14/15: n=20, N=7). All means ± s.e.m (Supplementary Table 3). n=number of cells, N=number of mice.

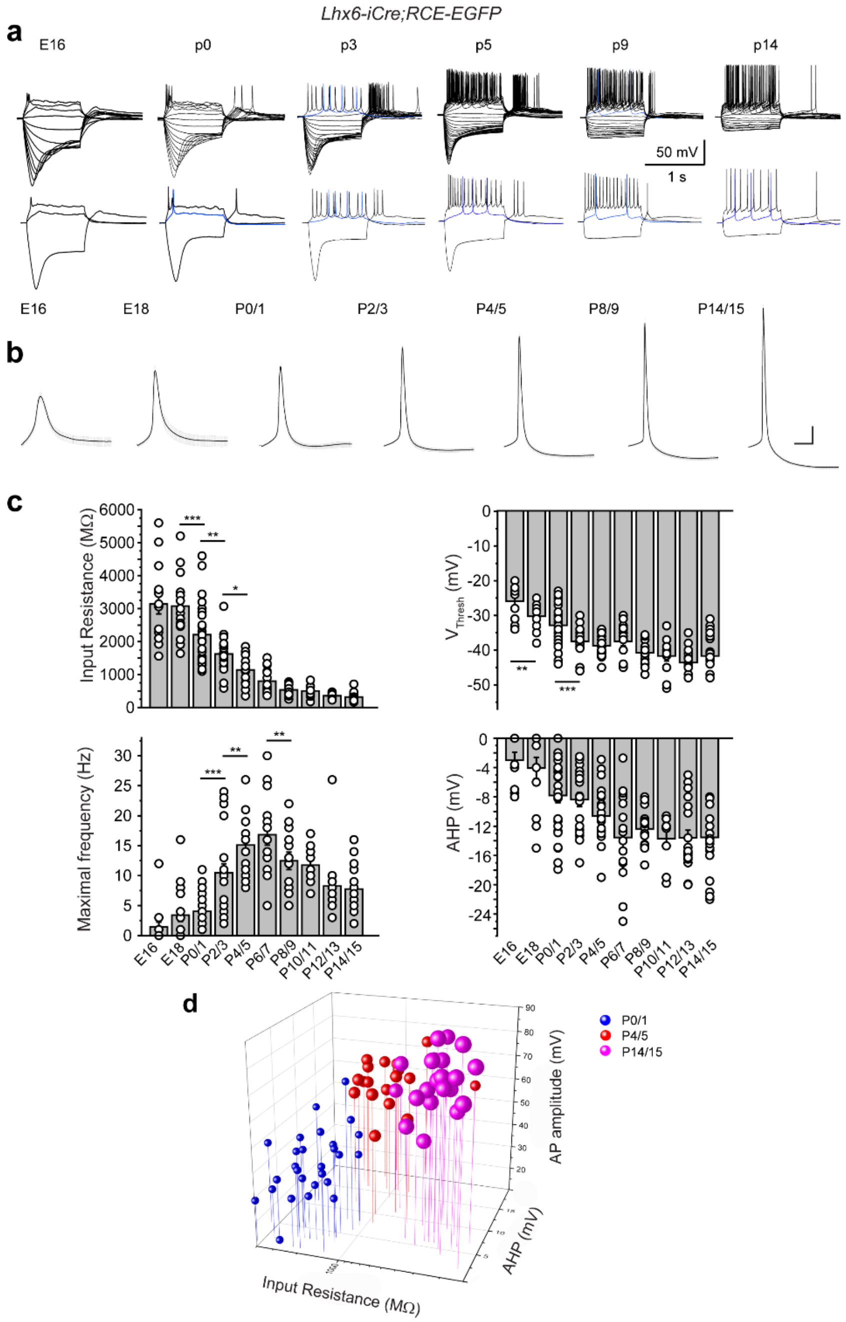

Supplementary Fig. 4: Development of CGINs electrophysiological properties (E16-P15). a (top), Representative whole-cell voltage responses to a series of current pulses. Maximal hyperpolarizing pulse for E16: -40 pA; P0: -50 pA; for P3, P5, P9 and P15: -100 pA, increments: for E16-P3: 5 pA for P5-P15: 10 pA. **a (**bottom**),** traces induced by maximal hyperpolarizing or depolarizing pulses or by a depolarizing pulse at threshold (blue)**. b**, Averaged action potential waveform for indicated ages (E16: n=22, N=7; E18: n=17, N=6; P0/1: n=30, N=8; P2/3: n= 22, N=7; P4/5: n=20, N=6; P8/9: n=17, N=4; P14/15: n=22, N=6. **c**, Quantification of the main intrinsic membrane properties of CGINs during development. AHP: afterhyperpolarization, V_{Thresh}: action potential threshold. **c**, 3-D plot of intrinsic physiological properties at the indicated ages. (E16: n=22, N=7; E18: n=17, N=6; P0/1: n=30, N=8; P2/3: n= 22, N=7; P4/5: n=20, N=6; P6/7: n=17, N=4; P8/9: n=17, N=4; P10/11: n= 12, N=4; P12/13: n=20, N=4; P14/15: n=22, N=6. All means ± s.e.m (Supplementary Table 4). n=number of cells, N=number of mice.

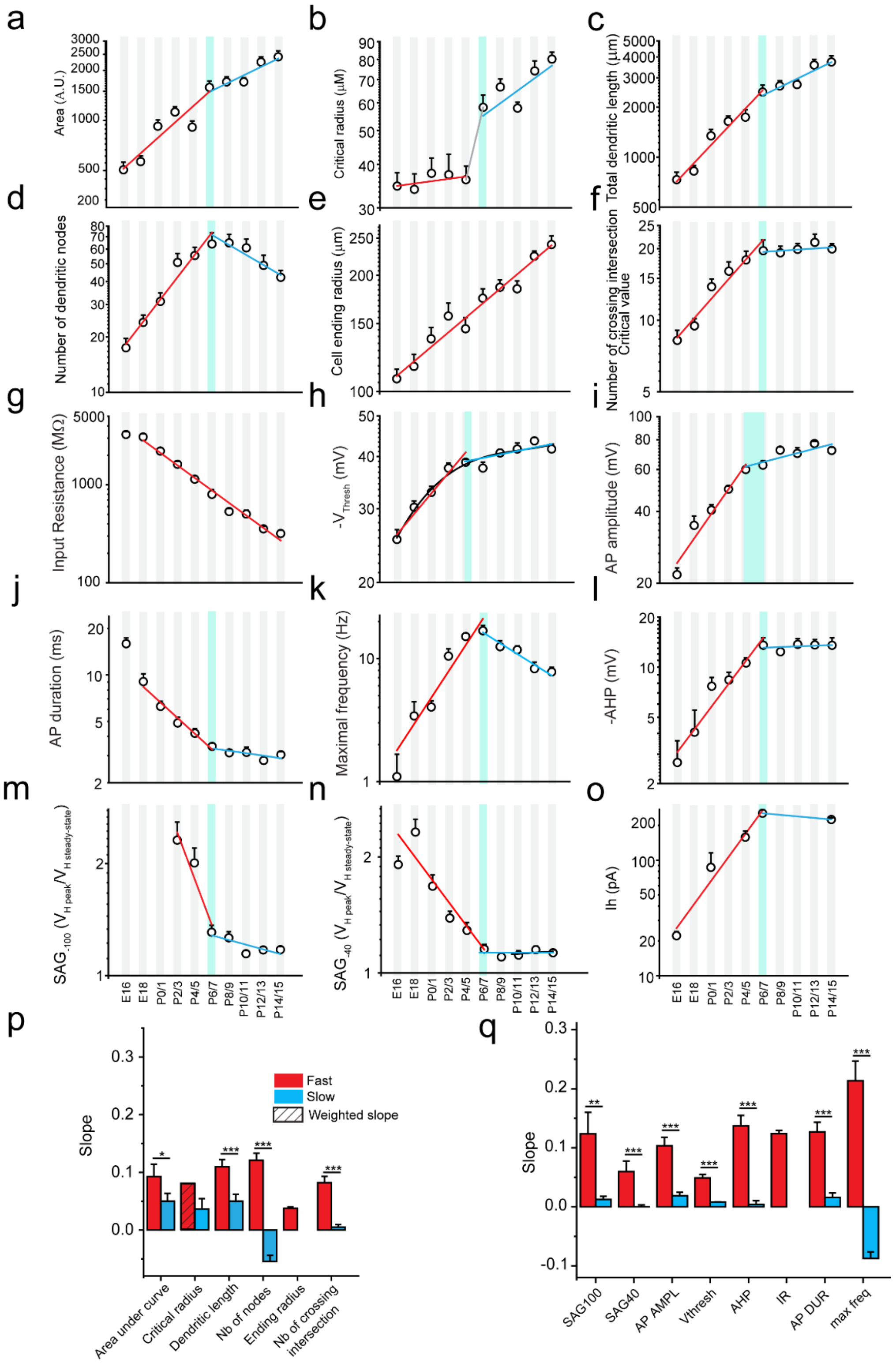

Supplementary Fig. 5: Rates of morphological and electrophysiological CGINs parameters changes during development (semi-log plots) (E16-P15).

a-o, Linear fits of fast (red) and slow (blue) changes of the CGINs intrinsic parameters shown in Fig. 1, Supplementary Figs 3, 4, 6, but in semi-log Y coordinates. Linear fits for morphological **(a-f)**, and electrophysiological **(g-o)** intrinsic parameters (for critical radius weighted fast slope is shown (**b**)). Vertical blue lines indicate the days of slope changes from fast to slow. **p, q,** Quantification of fast and slow rates. All means ± s.e.m (Supplementary Table 6).

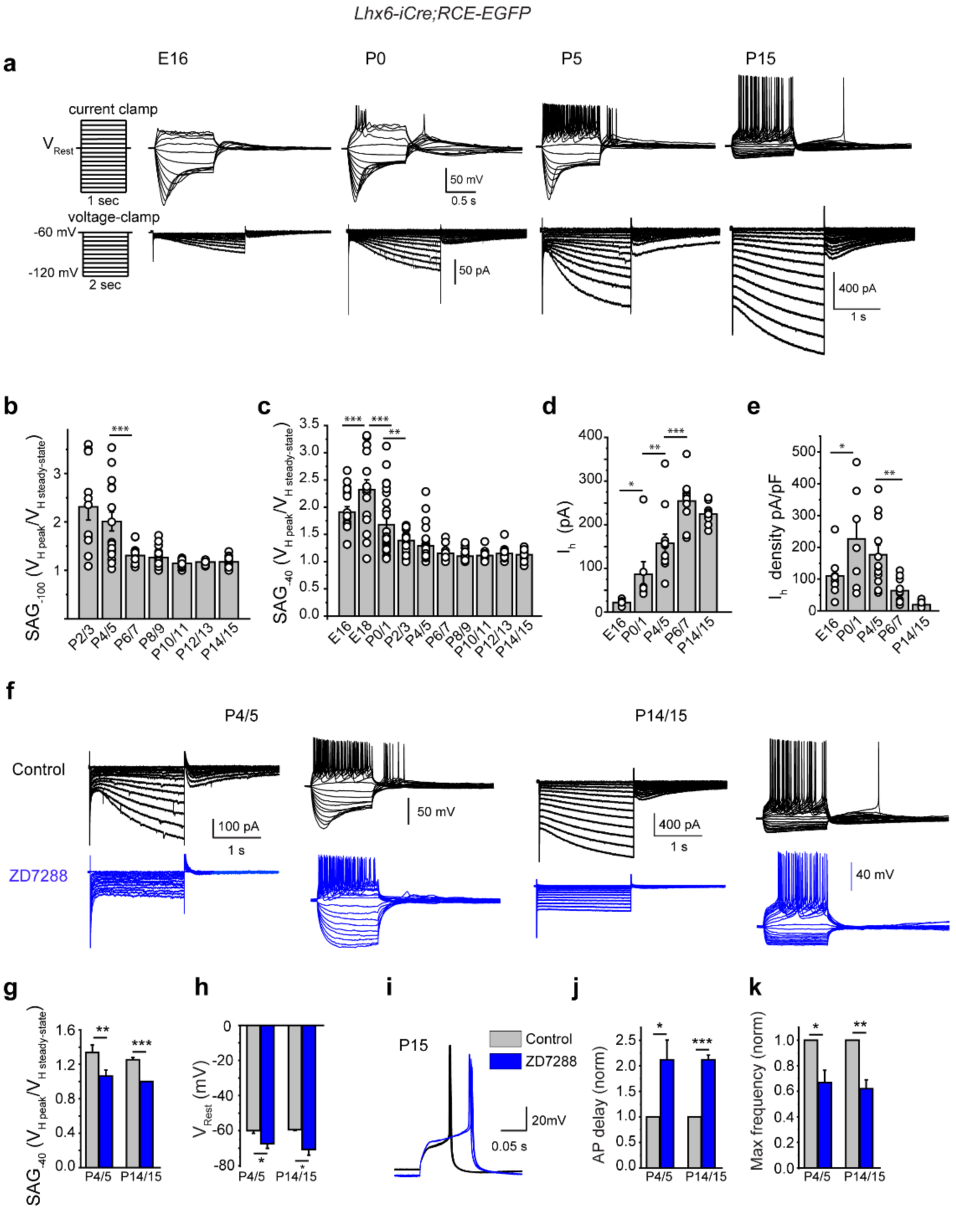

Supplementary Fig. 6: Development of the CGIN hyperpolarization-activated, cyclic nucleotide sensitive H current, I_h (E16-P15).

a (left), experimental protocols; **a**, representative voltage responses (top) and I_h currents (bottom) to 1-sec current pulses or 2-sec voltage steps correspondingly. Maximal hyperpolarizing current pulse at E16: -40 pA; P0: -50 pA; P5, P15: -100 pA, increments for E16-P0: 5 pA; P5, P15: 10 pA; Voltage steps from -60 to -120 mV, increments: 5 mV; holding potential V_H = -60 mV. **b**,**c**, Mean amplitude of the voltage sag evoked by a -100 pA current pulse or **c**, by a -40 pA current pulse (E16: n=22, N=7; E18: n=17, N=6; P0/1: n=30, N=8; P2/3: n= 22, N=7; P4/5: n=20, N=6; P6/7: n=17, N=4; P8/9: n= 17, N=4; P10/11: n= 12, N=4; P12/13: n= 20, N=4; P14/15: n=22, N=6. **d**, Mean I_h amplitude and **e**, Mean I_h density, as a function of age. (E16: n=8, N=3; P0/1: n=7, N=3; P4/5: n=12, N=3; P7: n=10, N=3; P14/15: n= 11, N=3**.** **f**, Representative traces for I_h (left traces) or sag (right traces) recorded from the same cells before (black) and after application of ZD7288 (100 µM, blue) at P4/5 and P14/15. **g-k**, Quantification of ZD7288 effects: **g**, Mean SAG$_{-40}$ amplitude, **h**, Mean resting membrane potential (V_{Rest}), **i**-representative traces of AP, in control (grey) and in the presence of ZD7288 (blue). **j**, Mean latency of the first evoked spike normalized to control, **k**, Mean maximal spike frequency normalized to control. (P4/5 control: n=5, N=3; P4/5 ZD7288: n=5, N=3; P14/15 control: n=5, N=3; P14/15 ZD7288: n=5, N=3). All means ± s.e.m (Supplementary Table 5). n=number of cells, N=number of mice.

Lhx6-iCre;RCE-EGFP

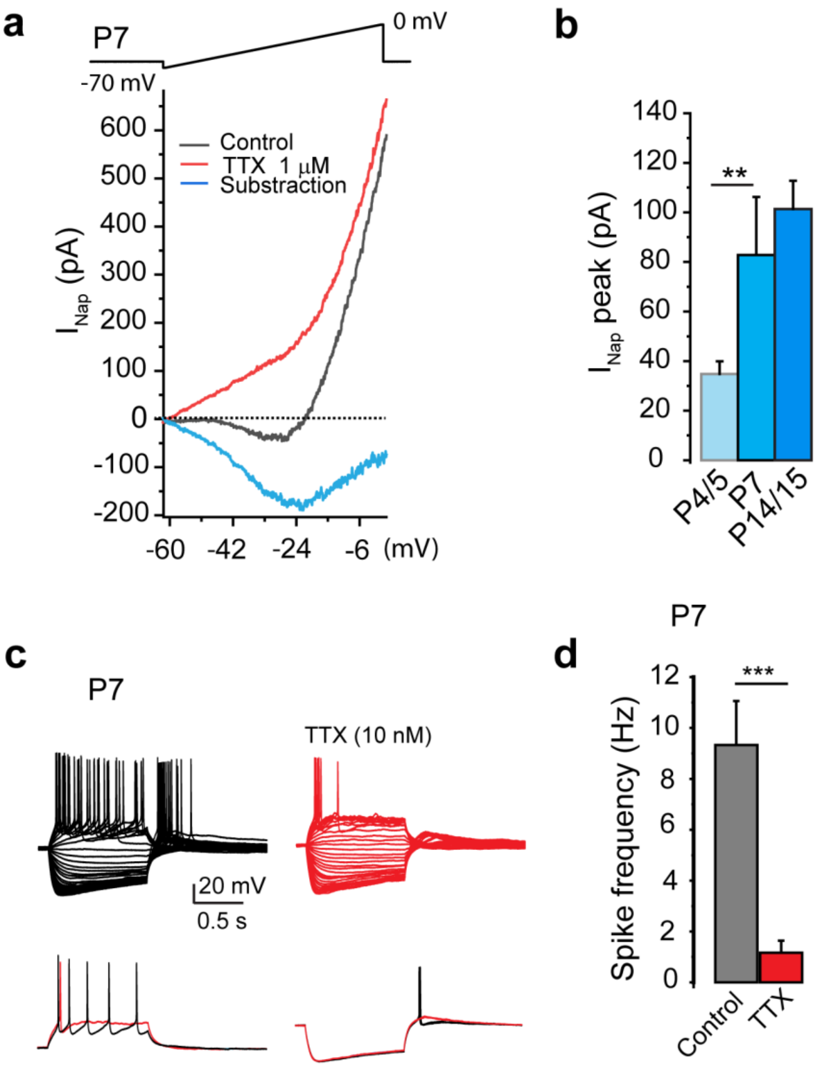

Supplementary Fig. 7: Postnatal development of the CGIN persistent sodium current, I_{NaP} (P5-P15). **a**, (top): experimental protocol (ramp stimulation from -70 mv to 0 mV); **(**bottom**)** representative current traces averaged from five superimposed traces evoked by a 2-sec voltage ramp before (black) and during TTX (1 µM) application (red). The I_{NaP} trace (blue) was obtained by subtraction of the TTX-resistant current (red) from the total current (black). **b**, Mean I_{NaP} peak amplitudes at P4/5, P7, P14/15 (P4/5: n= 9, N=4; P7: n= 7, N=3; P14/15: n= 6, N=3. **c**, Representative traces of voltage responses to 1-sec current injections at P7 in control (black) and in the presence of TTX (10 nM, red); (bottom): selected traces from c top, induced by maximal hyperpolarizing and depolarizing current pulses. **d**, Mean maximal evoked spike frequency before and during TTX (10 nM) application (P7 control: n= 6, N=3; P7 TTX: n= 6, N=3. All means ± s.e.m (Supplementary Table 7). n=number of cells, N=number of mice.

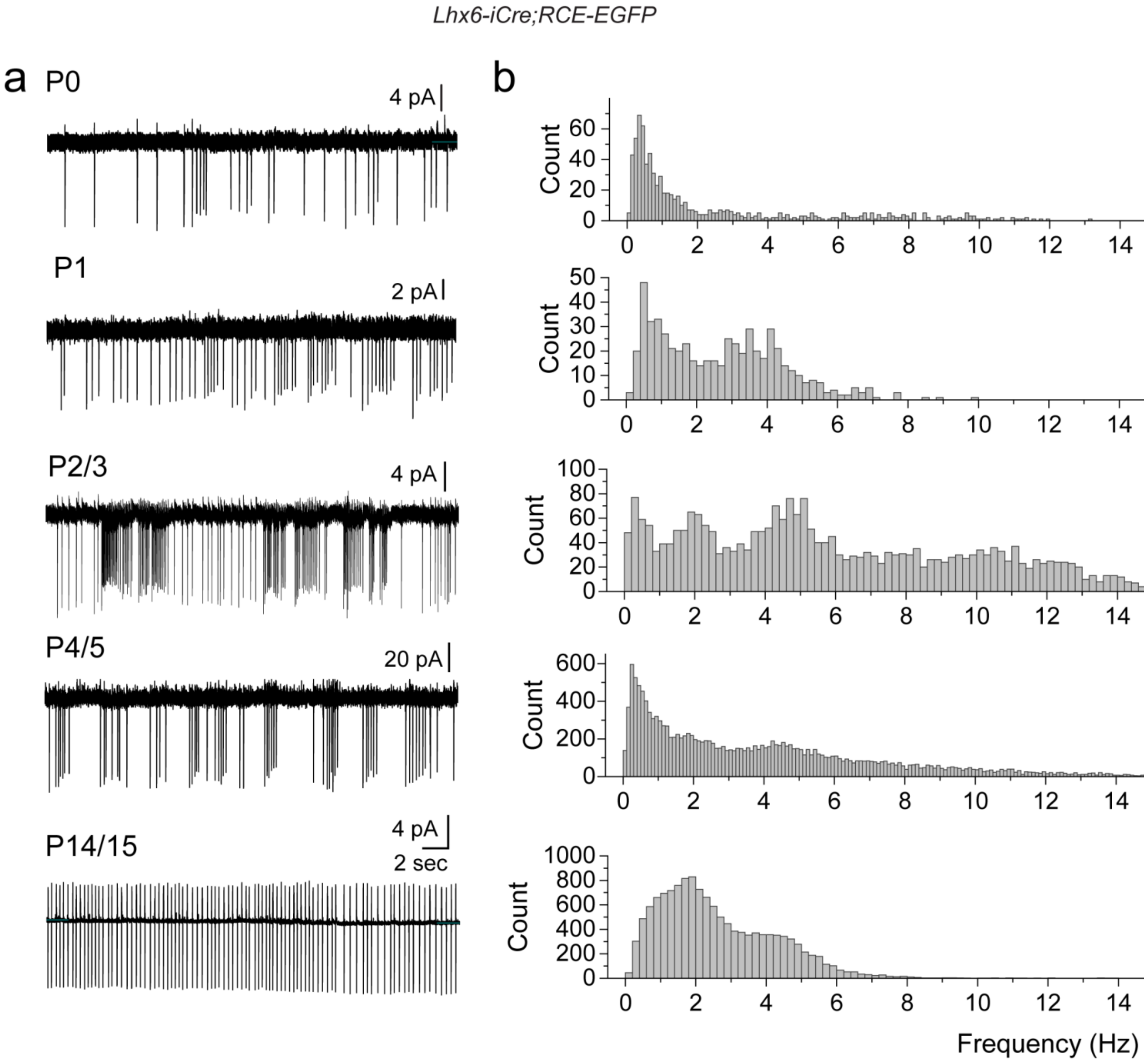

Supplementary Fig. 8: Patterns of CGINs ongoing activity during early postnatal development (P0-P15)
a, Representative cell-attached recordings of CGINs ongoing activity at the indicated ages. **b**, Pooled instantaneous frequency distributions of CGINs ongoing activity recorded in cell-attached configuration at the same ages (P0: n=11, N=4, 768 events; P1: n=6, N=3, 577 events; P2/3: n=13, N=3, 2680 events; P4/5: n=43, N=18; 15987 events; P14/15: n=24, N=10, 13504 events). n=number of cells, N=number of mice.

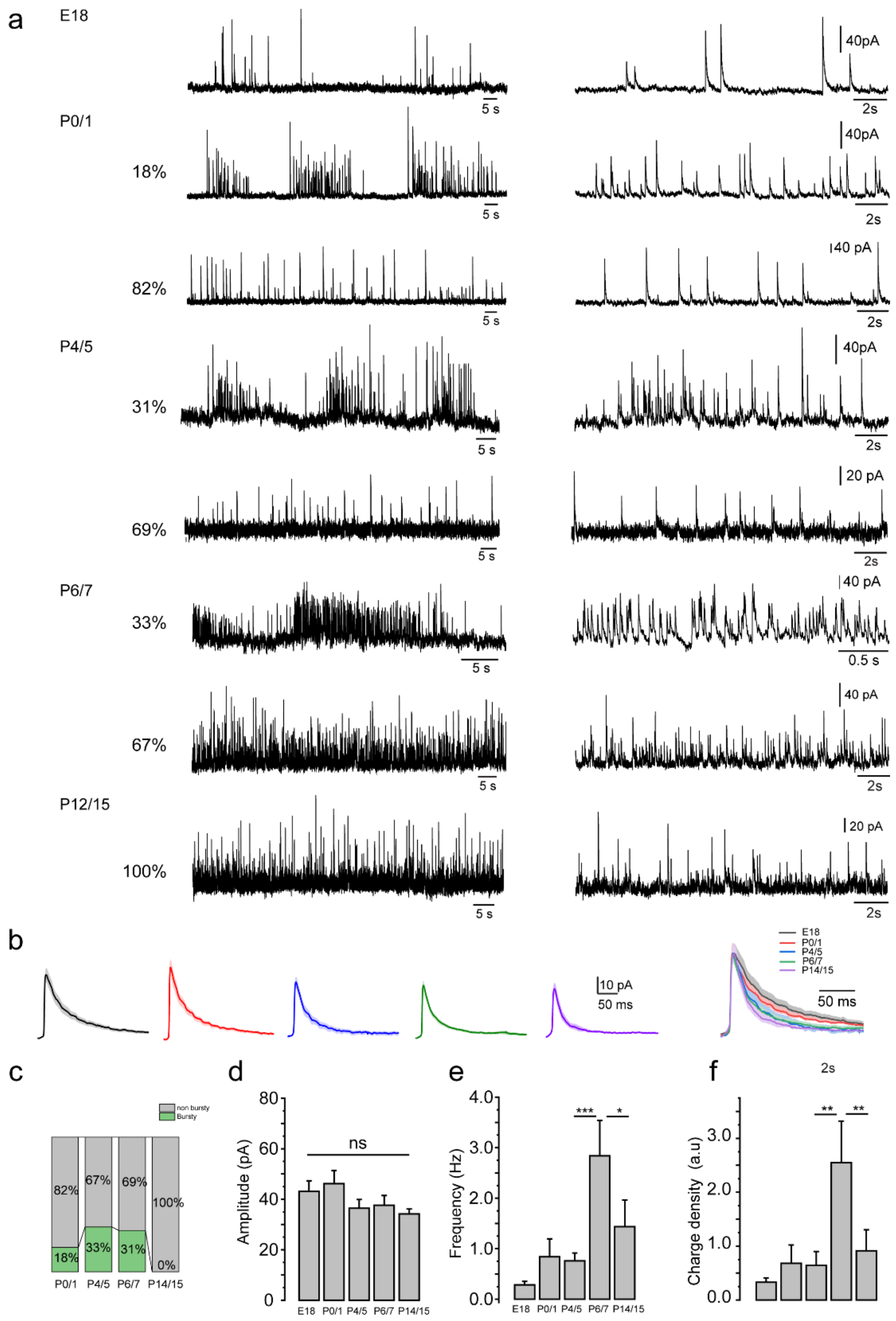

Supplementary Fig. 9: Development of spontaneous GABA$_A$R-mediated activity recorded from CGINs (E18-P15).

a, Representative traces of spontaneous GABAergic PSCs (voltage-clamp recordings, V_H = +10 mV) at the indicated ages. (right), recordings at an extended time scale. **b,** Averaged traces of spontaneous GABAergic PSCs at the same ages as in **(a)**. **c,** Percent of cells displaying a "bursting" pattern as a function of age. **d,** Mean amplitude, **e,** Mean frequency, and **f,** Mean charge density, of GABAergic PSCs (E18: n=9, N=5; P0/1: n=9, N=5; P4/5: n= 10, N=8; P6/7: n= 9, N=4; P14/15: n= 9, N=5 (Supplementary Table 10). n=number of cells, N=number of mice.

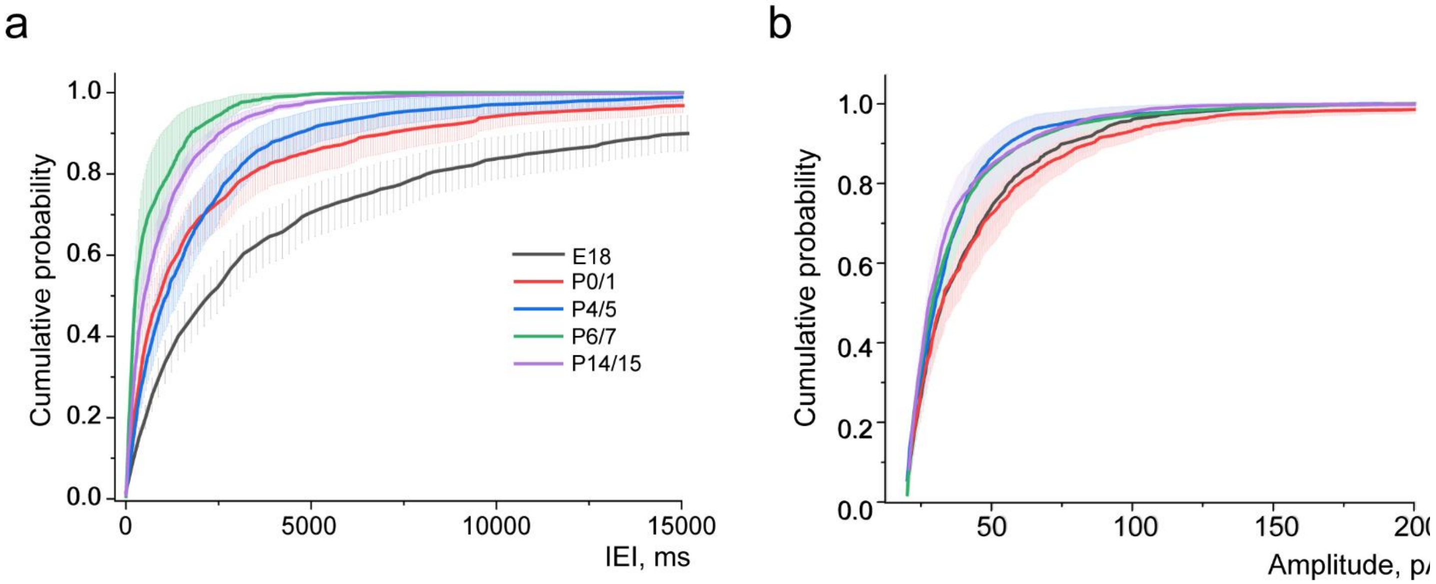

Supplementary Fig. 10: **Frequency and amplitude of spontaneous $GABA_A$R-mediated PSCs recorded from CGINs (E18-P15).**

a, Cumulative probability of inter-event intervals (IEI). **b,** Cumulative probability of spontaneous GABAergic PSCs amplitude. From the same data set as in Supplementary Fig. 9.

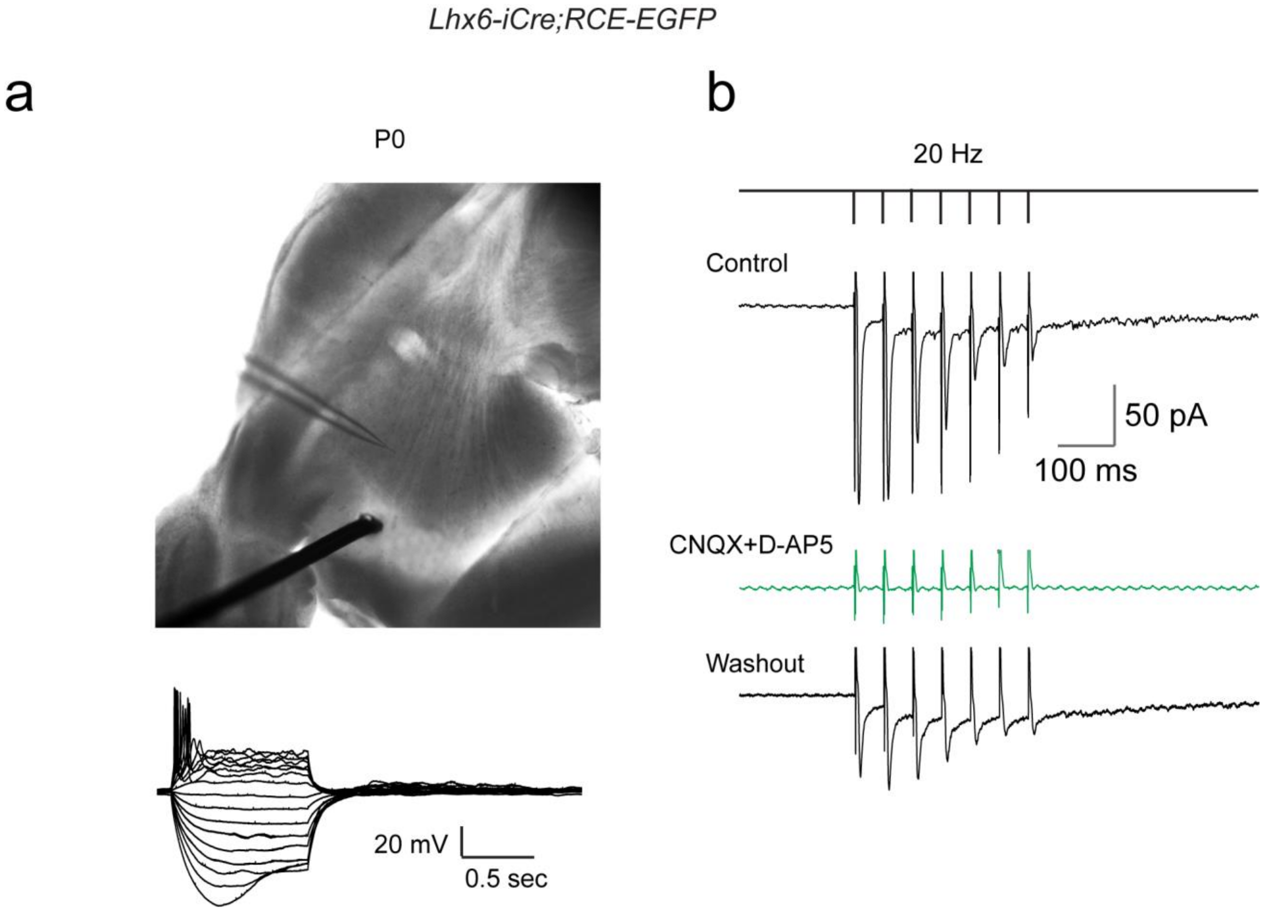

Supplementary Fig. 11: Cortico-striatal glutamatergic EPSCs evoked in CGINS at P0
a top, photo of a sagittal slice showing the location of the extracellular stimulating electrode in the cortex and of the recording pipette in the dorsal striatum. **a** bottom, Typical whole-cell CGIN voltage responses to a series of current pulses at P0. **b** top, Stimulation protocol; below are representative averaged (n=10) whole-cell EPSCs recorded at P0 in response to cortical stimuli in control ACSF, during bath application of CNQX (20 µM) and D-AP5 (50 µM) and after their wash-out (n=3 cells).

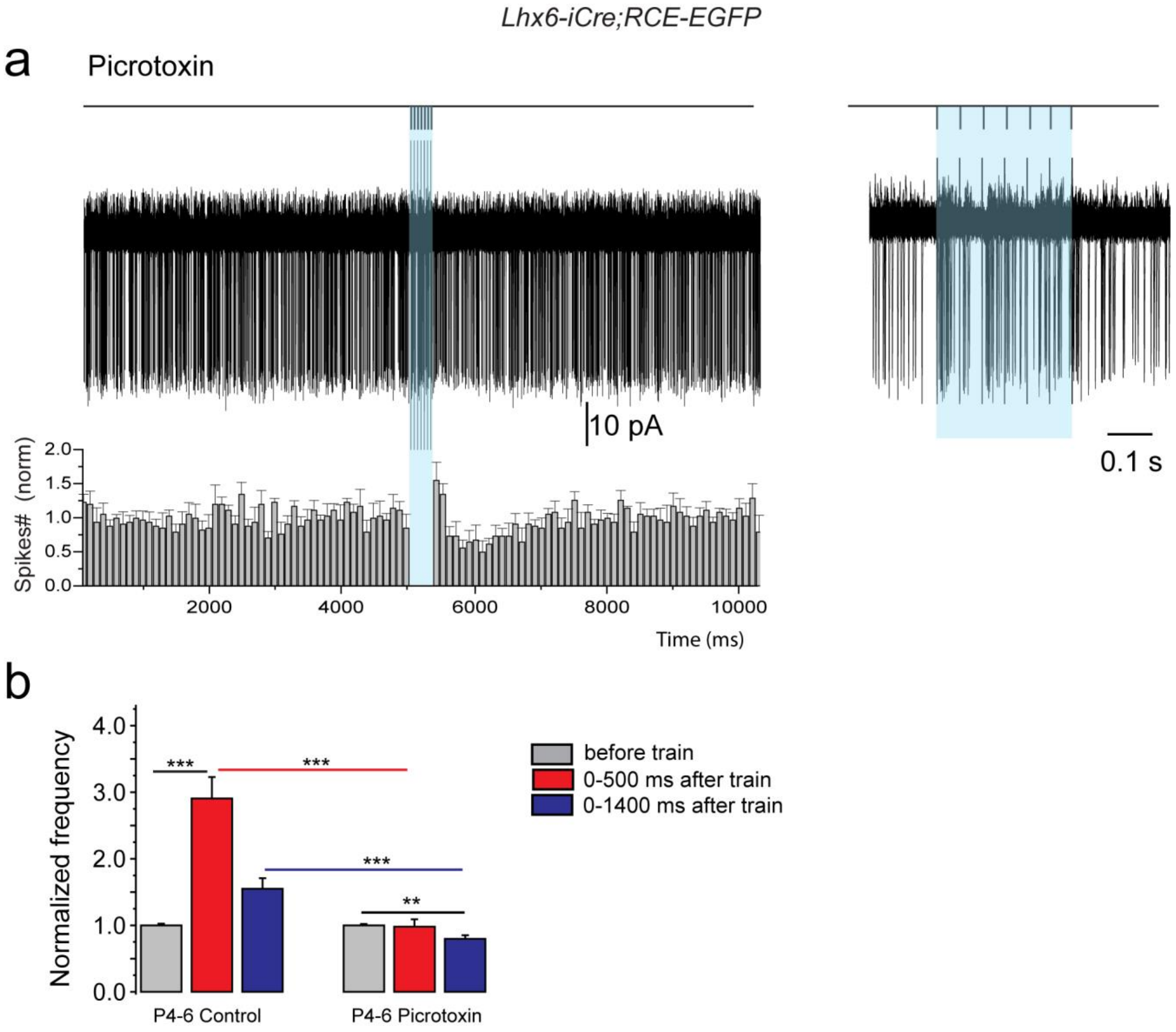

Supplementary Fig. 12: Picrotoxin-sensitivity of the excitatory response of CGINs to cortical stimulation in striatal slices from control pups (P4-6).

a, (from top to bottom), Cortical stimulation protocol (20 Hz, 7 stimuli); Bath application of picrotoxin (50 µM) totally blocked the polysynaptic (post-train) excitatory response to cortical stimulation (representative superimposed cell-attached recordings, n=30) and mean frequency histograms (control: n=8 cells, N=4; picrotoxin: 6 cells, N=3); right: responses within the train at extended time scale showing the reliability of cortical input to the recorded CGIN. **b**, Mean number of spikes during two different time windows after train stimulation (0–500 ms, 0–1400 ms) normalized to spikes counts before train: P4-6 control excitatory response as in Fig. 4 vs P4-6 picrotoxin. All means ± s.e.m (Supplementary Table 12). n=number of cells, N=number of mice.

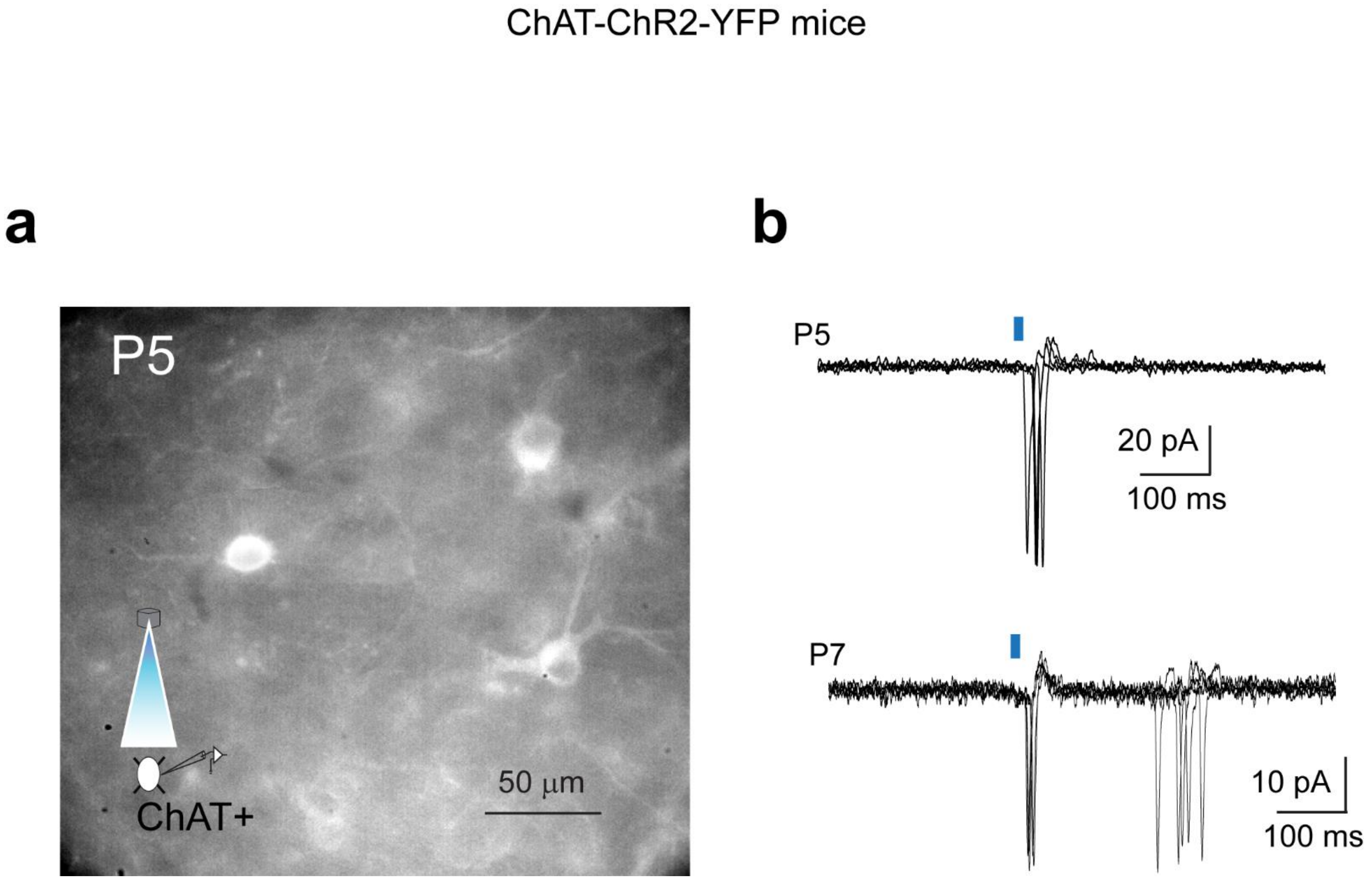

Supplementary Fig. 13: Optical control of the spatiotemporal neuronal activity of cholinergic interneurons using a digital micro-mirror device (DMD). a, Fluorescent image of YFP+/ channelrhodopsin-2 (ChR2) expressing cells in the dorsal striatum (sagittal slice from a ChAT-ChR2-YFP mouse). ChAT+-ChR2^{+} presynaptic axonal terminals were activated in a 250 µm x 250 µm field. **b**, A brief light pulse (490 nm, 20-30 ms, vertical blue bars) directly triggered action potentials in ChR2-expressing neuron (superimposed 5 consecutive cell-attached recordings) with millisecond temporal precision at P5 and P7.

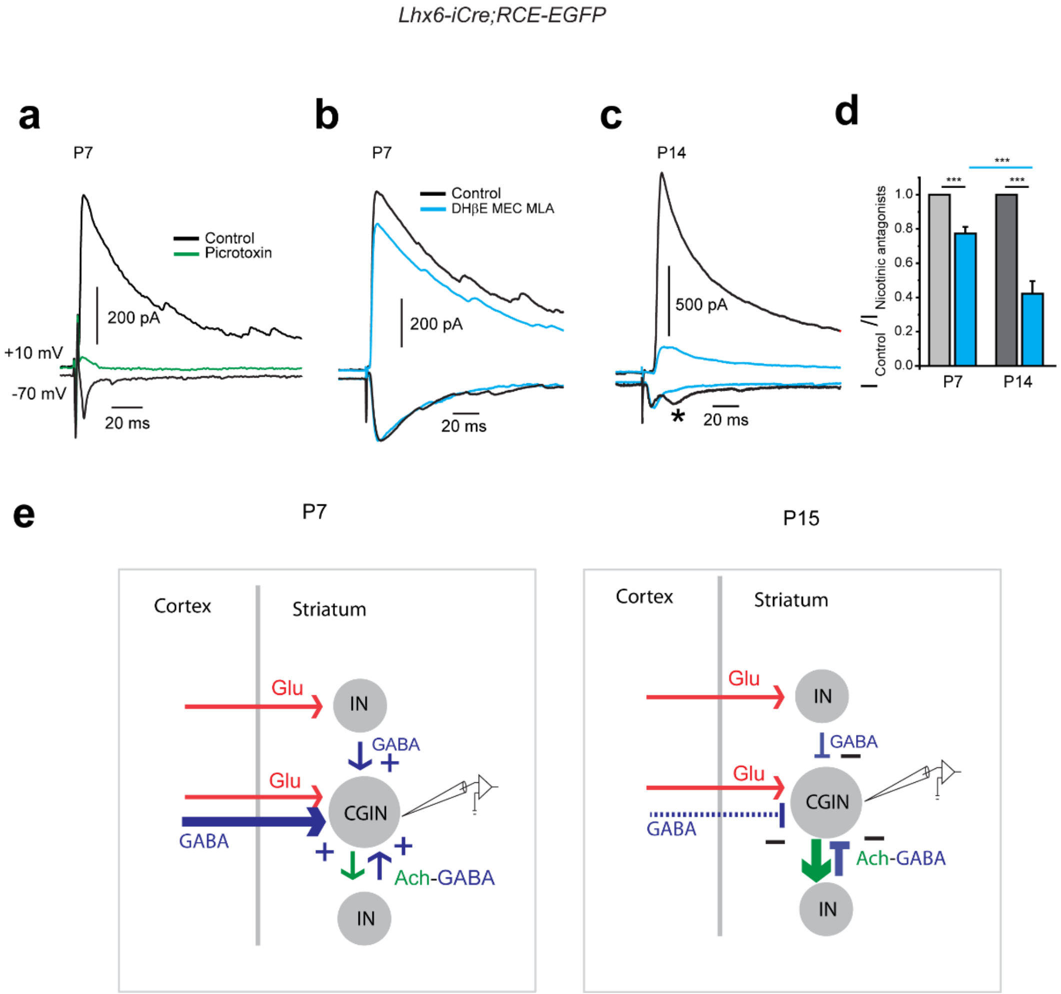

Supplementary Fig.14: Age-dependent GABAergic component of the polysynaptic microcircuit from cortex to CGINs

a, Cortical stimulation reliably evoked in the recorded CGIN a (glutamatergic) inward PSC (black) at V_H = -70 mV and an outward PSC at V_H = +10 mV (black). At P7, bath application of picrotoxin (50 µM) suppressed a large part of the outward PSC (green) showing that it was mainly mediated by $GABA_A$ receptors. **b,c,** Nicotinic receptor antagonists differently inhibited the GABAergic outward PSCs at P7 (b) and P14 (c) but did not affect the glutamatergic inward PSC. Note that stimulation at V_H = - 70 mV evoked a large inward glutamatergic PSC at P14 but a smaller one followed by a second nicotinic antagonists-sensitive PSC (asterisk). Since CNQX (10 µM) blocked both PSCs, the second PSC could reflect the appearance around P14 of an amplification mechanism of cortical excitatory inputs via the activation of presynaptic nAChRs on glutamatergic terminals, as recently described for spiny projection neurons[51]. **d,** Mean inhibition of the GABAergic PSC by nicotinic antagonists at P7 and P14/15 (P7: n= 9, N=3; P14/15: n= 9, N=3). **e**, Schematic representation of cortico-striatal microcircuits and of the putative weights (represented by the thickness of connections) of the different synapses at P7 and P15 (arrows: excitation, bars: inhibition, see text for explanations). All means ± s.e.m. (Supplementary Table 14). n=number of cells, N=number of mice.

SUPPLEMENTARY TABLES

Supplementary table 1

Statistical analysis (significant differences are highlighted in blue)

Supplementary Fig. 1. Identification of the time window between birth and walking acquisition.

Supplementary Fig. 1b

One-way ANOVA Kruskal-Wallis test with Dunn's Multiple comparison post-hoc test

Groups	n (pups)	N (litter)	Number of ultrasonic calls Mean ± SEM
**P2/3**	63	7	110.43 ± 9.34
**P4/5**	63	7	174.90 ± 14.49
**P6/7**	63	7	173.79± 16.50
**P8/9**	54	6	115.43 ± 16.28
**P10/11**	54	6	23.43 ± 10.90
**P12/13**	62	7	11.38 ± 4.54
**P14/15**	61	7	1.344 ± 0.59

groups	Kruskall-Wallis test p value	Dunn's adj. p value
**P2/3 vs. P4/5**	< 0.0001	> 0,9999
**P2/3 vs. P6/7**		> 0,9999
**P2/3 vs. P8/9**		> 0,9999
**P2/3 vs. P10/11**		< 0,0001
**P2/3 vs. P12/13**		< 0,0001
**P2/3 vs. P14/15**		< 0,0001
**P4/5 vs. P6/7**		> 0,9999
**P4/5 vs. P8/9**		0.5486
**P4/5 vs. P10/11**		< 0,0001
**P4/5 vs. P12/13**		< 0,0001
**P4/5 vs. P14/15**		< 0,0001
**P6/7 vs. P8/9**		> 0,9999
**P6/7 vs. P10/11**		< 0,0001
**P6/7 vs. P12/13**		< 0,0001
**P6/7 vs. P14/15**		< 0,0001
**P8/9 vs. P10/11**		< 0,0001
**P8/9 vs. P12/13**		< 0,0001
**P8/9 vs. P14/15**		< 0,0001
**P10/11 vs. P12/13**		> 0,9999
**P10/11 vs. P14/15**		0.3406
**P12/13 vs. P14/15**		0.4002

Supplementary Fig. 1d
One-way ANOVA Kruskal-Wallis test with Dunn's Multiple comparison post-hoc test

Maximal number of pups per cluster

Groups	n (pups)	N (litter)	Maximum Number of Pups per Cluster Mean ± SEM
**P2/3**	63	7	2.00 ± 0.38
**P4/5**	54	6	2.17 ± 0.55
**P6/7**	54	6	2.00 ± 0.45
**P8/9**	54	6	3.17 ± 0.31
**P10/11**	72	8	4.88 ± 0.588
**P12/13**	63	7	4.43 ± 0.43
**P14/15**	63	7	5.43 ± 0.75

Maximal Number of Pups per Cluster Groups	Kruskall-Wallis test p value	Dunn's adj. p value
**P2/3 vs. P4/5**	< 0.0001	> 0,9999
**P2/3 vs. P6/7**		> 0,9999
**P2/3 vs. P8/9**		> 0,9999
**P2/3 vs. P10/11**		0.0217
**P2/3 vs. P12/13**		0.0619
**P2/3 vs. P14/15**		0.0091
**P4/5 vs. P6/7**		> 0,9999
**P4/5 vs. P8/9**		> 0,9999
**P4/5 vs. P10/11**		0.0925
**P4/5 vs. P12/13**		0.2144
**P4/5 vs. P14/15**		0.0415
**P6/7 vs. P8/9**		> 0,9999
**P6/7 vs. P10/11**		0.0422
**P6/7 vs. P12/13**		0.1065
**P6/7 vs. P14/15**		0.0184
**P8/9 vs. P10/11**		> 0,9999
**P8/9 vs. P12/13**		> 0,9999
**P8/9 vs. P14/15**		> 0,9999
**P10/11 vs. P12/13**		> 0,9999
**P10/11 vs. P14/15**		> 0,9999
**P12/13 vs. P14/15**		> 0,9999

Number of Clusters During 10 min Recording

Groups	n (pups)	N (litter)	Number of Clusters During 10 min Recording Mean ± SEM
**P2/3**	63	7	0.29 ± 0.18
**P4/5**	54	6	0.50 ± 0.3
**P6/7**	54	6	0.33 ± 0.21
**P8/9**	54	6	1.17 ± 0.31
**P10/11**	72	8	1.88 ± 0.30
**P12/13**	63	7	3.00 ± 0.53

**P14/15**	63	7	2.714 ± 0.97

Number of Clusters During 10 min Recording<br>Groups	Kruskall-Wallis test p value	Dunn's adj. p value
**P2/3 vs. P4/5**	< 0.0001	> 0,9999
**P2/3 vs. P6/7**		> 0,9999
**P2/3 vs. P8/9**		> 0,9999
**P2/3 vs. P10/11**		0.0811
**P2/3 vs. P12/13**		0.0041
**P2/3 vs. P14/15**		0.0742
**P4/5 vs. P6/7**		> 0,9999
**P4/5 vs. P8/9**		> 0,9999
**P4/5 vs. P10/11**		0.3625
**P4/5 vs. P12/13**		0.0287
**P4/5 vs. P14/15**		0.3202
**P6/7 vs. P8/9**		> 0,9999
**P6/7 vs. P10/11**		0.1582
**P6/7 vs. P12/13**		0.0104
**P6/7 vs. P14/15**		0.1416
**P8/9 vs. P10/11**		> 0,9999
**P8/9 vs. P12/13**		> 0,9999
**P8/9 vs. P14/15**		> 0,9999
**P10/11 vs. P12/13**		> 0,9999
**P10/11 vs. P14/15**		> 0,9999
**P12/13 vs. P14/15**		> 0,9999

Number of Cluster Switches

Groups	n (pups)	N (litter)	Number of Cluster Switches<br>Mean ± SEM
**P2/3**	63	7	0 ± 0
**P4/5**	54	6	0 ± 0
**P6/7**	54	6	0 ± 0
**P8/9**	54	6	0.17 ± 0.17
**P10/11**	72	8	2.63 ±1.13
**P12/13**	63	7	2.29 ± 0.68
**P14/15**	63	7	7.43 ± 4.08

Number of Cluster Switches<br>Groups	Kruskall-Wallis test p value	Dunn's adj. p value
**P2/3 vs. P4/5**	< 0.0001	> 0,9999
**P2/3 vs. P6/7**		> 0,9999
**P2/3 vs. P8/9**		> 0,9999
**P2/3 vs. P10/11**		0.0354
**P2/3 vs. P12/13**		0.0131
**P2/3 vs. P14/15**		0.232
**P4/5 vs. P6/7**		> 0,9999
**P4/5 vs. P8/9**		> 0,9999
**P4/5 vs. P10/11**		0.0548

**P4/5 vs. P12/13**		0.0213
**P4/5 vs. P14/15**		0.3071
**P6/7 vs. P8/9**		> 0,9999
**P6/7 vs. P10/11**		0.0548
**P6/7 vs. P12/13**		0.0213
**P6/7 vs. P14/15**		0.3071
**P8/9 vs. P10/11**		0.2161
**P8/9 vs. P12/13**		0.0903
**P8/9 vs. P14/15**		0.9327
**P10/11 vs. P12/13**		> 0,9999
**P10/11 vs. P14/15**		> 0,9999
**P12/13 vs. P14/15**		> 0,9999

Supplementary table 2

Statistical analysis (significant differences are highlighted in blue)

Fig. 1: Development of CGINs intrinsic properties from E16 to P15: a summary
Fig. 1 c, d
One-way ANOVA Kruskal-Wallis test with Dunn's Multiple comparison post-hoc test

Groups	n (cells)	N (mice)	Sholl area under curve (A.U.) Mean ± SEM	Critical radius (µm) Mean ± SEM
**E16**	21	5	505.29 ± 55.56	34.65 ± 3.18
**E18**	21	3	566.05 ± 45.87	33.90 ± 3.67
**P0/1**	20	6	923.46 ± 84.69	37.69 ± 3.97
**P2/3**	20	7	1124.46 ± 82.66	37.36 ± 5.39
**P4/5**	20	13	911.21 ± 82.77	36.14 ± 3.36
**P6/7**	21	4	1580.38 ± 146.82	58.26 ± 4.95
**P8/9**	20	5	1705.91 ± 125.73	66.64 ± 3.64
**P10/11**	21	12	1705.90 ± 101.73	57.95 ± 2.32
**P12/13**	21	5	2245.57 ± 166.84	74.12 ± 5.08
**P14/15**	20	7	2412.45 ± 210.57	80.13 ± 3.70

Sholl area under curve (A.U.) groups	Kruskall-Wallis test p value	Dunn's adj. p value
**E16 vs E18**	< 0.0001	> 0.9999
**E18 vs P0/1**		0.5364
**P0/1 vs P2/3**		> 0.9999
**P2/3 vs P4/5**		> 0.9999
**P4/5 vs P6/7**		0.0349
**P6/7 vs P8/9**		> 0.9999
**P8/9 vs P10/11**		> 0.9999
**P10/11 vs P12/13**		> 0.9999
**P12/13 vs P14/15**		> 0.9999

Critical radius (µm) groups	Kruskall-Wallis test p value	Dunn's adj. p value
**E16 vs E18**	< 0.0001	> 0.9999
**E18 vs P0/1**		> 0.9999
**P0/1 vs P2/3**		> 0.9999

**P2/3 vs P4/5**		> 0.9999
**P4/5 vs P6/7**		0.0256
**P6/7 vs P8/9**		> 0.9999
**P8/9 vs P10/11**		> 0.9999
**P10/11 vs P12/13**		0.8047
**P12/13 vs P14/15**		> 0.9999

Fig. 1e,f,g
One way ANOVA with Fisher's LSD post-hoc test

Groups	n (cells)	N (mice)	AP amplitude (pA) Mean ± SEM	n (cells)	N (mice)	AP duration (ms) Mean ± SEM	n (cells)	N (mice)	Mean sag amplitude at -40 pA Mean ± SEM
**E16**	12	7	21.71 ± 1.46	9	7	15.95 ± 1.43	19	7	1.91 ± 0.09
**E18**	15	6	34.97 ± 3.28	10	6	9.07 ± 1.11	15	6	2.32 ± 0.18
**P0/1**	29	8	40.52 ± 2.13	26	8	6.25 ± 0.51	25	8	1.68 ± 0.12
**P2/3**	22	7	49.64 ± 1.50	22	7	4.88 ± 0.44	18	7	1.39 ± 0.06
**P4/5**	20	6	60.00 ±1.60	20	6	4.19 ± 0.29	25	6	1.29 ± 0.06
**P6/7**	17	4	62.59 ± 2.85	17	4	3.44 ± 0.18	17	4	1.15 ± 0.03
**P8/9**	17	4	72.35 ± 2.20	17	4	3.13 ± 0.12	17	4	1.10 ±0.03
**P10/11**	12	4	70.12 ± 3.85	12	4	3.15 ± 0.25	12	4	1.11 ±0.03
**P12/13**	20	4	77.12 ± 1.73	20	4	2.79 ± 0.15	17	4	1.15 ±0.03
**P14/15**	22	6	72.12 ± 2.28	20	6	3.04 ± 0.15	22	6	1.13 ±0.02

AP amplitude (pA) groups	Univariate ANOVA between groups	Post-hoc test between the groups
**E16 vs E18**	< 0.0001	$7*E^{-4}$
**E18 vs P0/1**		0.08
**P0/1 vs P2/3**		0.00136
**P2/3 vs P4/5**		$9*E^{-4}$
**P4/5 vs P6/7**		0.43
**P6/7 vs P8/9**		0.00454
**P8/9 vs P10/11**		0.55
**P10/11 vs P12/13**		0.054
**P12/13 vs P14/15**		0.10

AP duration (ms) groups	Univariate ANOVA between groups	Post-hoc test between the groups
**E16 vs E18**	< 0.0001	$4*E^{-13}$
**E18 vs P0/1**		$1*E^{-4}$
**P0/1 vs P2/3**		0.014
**P2/3 vs P4/5**		0.24
**P4/5 vs P6/7**		0.24
**P6/7 vs P8/9**		0.63
**P8/9 vs P10/11**		0.98
**P10/11 vs P12/13**		0.60
**P12/13 vs P14/15**		0.67

Mean amplitude of SAG (current injection) at 40 pA Groups	Univariate ANOVA between groups	Post-hoc test between the groups
**E16 vs E18**	$5*E^{-25}$	$1*E^{-3}$
**E18 vs P0/1**		$2*E^{-7}$
**P0/1 vs P2/3**		0.0098
**P2/3 vs P4/5**		0.38
**P4/5 vs P6/7**		0.23
**P6/7 vs P8/9**		0.67
**P8/9 vs P10/11**		0.93
**P10/11 vs P12/13**		0.79
**P12/13 vs P14/15**		0.86

Supplementary table 3

Statistical analysis (significant differences are highlighted in blue)

Supplementary Fig. 3: development of CGINs morphological development (E16-P15).

Supplementary Fig. 3 c-f

One-way ANOVA Kruskal-Wallis test with Dunn's Multiple comparison post-hoc test

Groups	n (cells)	N (mice)	Total dendritic length (µm) Mean ± SEM	Number of dendritic truncs Mean ± SEM	Number of dendritic nodes Mean ± SEM	Ending radius (µm) Mean ± SEM	Critical value (Nb of crossing intersections) Mean ± SEM
**E16**	21	5	731.36 ± 79.98	4.14 ± 0.43	17.47 ± 2.17	107.86 ± 6.23	8.23 ± 0.84
**E18**	21	3	824.40 ± 65.45	3.62 ± 0.25	24.00 ± 2.32	116.14 ± 8.65	9.47 ± 0.69
**P0/1**	20	6	1342.56 ± 125.05	4.15 ± 0.46	31.20 ± 3.56	136.95 ± 9.48	13.83 ± 1.08
**P2/3**	20	7	1640.01 ± 126.48	4.50 ± 0.36	50.80 ± 6.05	156.90 ± 12.63	16.06 ± 1.52
**P4/5**	20	13	1736.79 ± 190.90	4.80 ± 0.38	55.35 ± 5.97	145.40 ± 9.94	17.91 ± 1.58
**P6/7**	21	4	2462.49 ± 47.26	3.91 ± 0.38	63.95 ± 9.62	174.48 ± 10.12	19.60 ± 2.12
**P8/9**	20	5	2677.44 ± 204.06	3.75 ± 0.26	64.85 ± 7.20	186.25 ± 8.60	19.19 ± 1.29
**P10/11**	21	12	2729.62 ± 178.12	3.86 ± 0.23	60.95 ± 7.10	184.57 ± 8.85	19.85 ± 1.15
**P12/13**	21	5	3576.97 ± 287.49	4.19 ± 0.32	48.95 ± 6.50	224.24 ± 7.10	21.24 ± 1.77
**P14/15**	20	7	3731.19 ± 337.79	3.40 ± 0.17	42.15 ± 3.79	240.40 ± 12.99	19.93 ± 1.05

Total dendritic length (µm) groups	Kruskall-Wallis test p value	Dunn's adj. p value
**E16 vs E18**	< 0.0001	> 0.9999
**E18 vs P0/1**		0.5734
**P0/1 vs P2/3**		> 0.9999
**P2/3 vs P4/5**		> 0.9999
**P4/5 vs P6/7**		0.6895
**P6/7 vs P8/9**		> 0.9999
**P8/9 vs P10/11**		> 0.9999
**P10/11 vs P12/13**		> 0.9999
**P12/13 vs P14/15**		> 0.9999

Number of dendritic truncs	Kruskall-Wallis test p value	Dunn's adj. p value

groups		
**E16 vs E18**	0.1665	> 0.9999
**E18 vs P0/1**		> 0.9999
**P0/1 vs P2/3**		> 0.9999
**P2/3 vs P4/5**		> 0.9999
**P4/5 vs P6/7**		0.3033
**P6/7 vs P8/9**		> 0.9999
**P8/9 vs P10/11**		> 0.9999
**P10/11 vs P12/13**		> 0.9999
**P12/13 vs P14/15**		0.7862

Number of dendritic nodes groups	Kruskall-Wallis test p value	Dunn's adj. p value
**E16 vs E18**	< 0.0001	> 0.9999
**E18 vs P0/1**		> 0.9999
**P0/1 vs P2/3**		0.1867
**P2/3 vs P4/5**		> 0.9999
**P4/5 vs P6/7**		> 0.9999
**P6/7 vs P8/9**		> 0.9999
**P8/9 vs P10/11**		> 0.9999
**P10/11 vs P12/13**		> 0.9999
**P12/13 vs P14/15**		> 0.9999

Ending radius (µm) groups	Kruskall-Wallis test p value	Dunn's adj. p value
**E16 vs E18**	< 0.0001	> 0.9999
**E18 vs P0/1**		> 0.9999
**P0/1 vs P2/3**		> 0.9999
**P2/3 vs P4/5**		> 0.9999
**P4/5 vs P6/7**		0.7130
**P6/7 vs P8/9**		> 0.9999
**P8/9 vs P10/11**		> 0.9999
**P10/11 vs P12/13**		0.3511
**P12/13 vs P14/15**		> 0.9999

Critical value groups	Kruskall-Wallis test p value	Dunn's adj. p value
**E16 vs E18**	< 0.0001	> 0.9999
**E18 vs P0/1**		0.2194
**P0/1 vs P2/3**		> 0.9999
**P2/3 vs P4/5**		> 0.9999
**P4/5 vs P6/7**		> 0.9999
**P6/7 vs P8/9**		> 0.9999
**P8/9 vs P10/11**		> 0.9999
**P10/11 vs P12/13**		> 0.9999
**P12/13 vs P14/15**		> 0.9999

Supplementary table 4

Statistical analysis (significant differences are highlighted in blue)

Supplementary Fig. 4: Development of CGINs electrophysiological properties (E16-P15).

Supplementary Fig. 4 c

One way ANOVA with Fisher's LSD post-hoc test (for data sets: Input resistance, Maximal Frequency and V_{Thresh}) and One-way ANOVA Kruskal-Wallis test with Dunn's Multiple comparison post-hoc test (for AHP)

Groups	n (cells)	N (mice)	Input resistance (MΩ) Mean ± SEM	Maximal frequency (Hz) Mean ± SEM	Vthresh (mV) Mean ± SEM	AHP (mV) Mean ± SEM
**E16**	22	7	3265.71 ± 228.81	1.09 ± 0.54	-25.33 ±1.41	-2.67 ± 0.94
**E18**	17	6	3078.24 ± 238.404	3.41 ± 1.04	-30.23 ±1.07	-4.07 ± 1.45
**P0/1**	30	8	2209.45 ± 177.475	4.03 ± 0.49	-32.83 ±1.08	-7.68 ± 1.02
**P2/3**	22	7	1622.05 ± 119.47	10.45 ± 1.53	-37.51 ±1.08	-8.38 ± 0.95
**P4/5**	20	6	1142.24 ± 97.39	15.1 ± 1.00	-38.76 ±0.61	-10.59 ± 0.79
**P6/7**	17	4	798.49 ± 94.10	16.82 ± 1.75	-37.52 ±1.27	-13.54 ± 1.50
**P8/9**	17	4	533.97 ± 38.18	12.47 ± 1.46	-40.78 ± 0.84	-12.38 ± 0.69
**P10/11**	12	4	503.42 ± 48.70	11.75 ± 0.93	-41.67 ±1.45	-13.71 ± 1.14
**P12/13**	20	4	355.69 ± 21.94	8.26 ± 1.07	-43.59 ±0.78	-13.59 ± 1.05
**P14/15**	22	6	318.38 ± 26.21	7.77 ± 0.71	-41.67 ±1.13	-13.53 ± 1.51

One way ANOVA with Fisher's LSD post-hoc test

Input resistance (MΩ groups	Univariate ANOVA between groups	Post-hoc test between the groups
**E16 vs E18**	< 0.0001 $3*E^{-46}$	0.38
**E18 vs P0/1**		$2*E^{-5}$
**P0/1 vs P2/3**		0.002
**P2/3 vs P4/5**		0.024
**P4/5 vs P6/7**		0.12
**P6/7 vs P8/9**		0.24
**P8/9 vs P10/11**		0.9
**P10/11 vs P12/13**		0.6
**P12/13 vs P14/15**		0.9

One way ANOVA with Fisher's LSD post-hoc test

Maximal frequency groups	Univariate ANOVA between groups	Post-hoc test between the groups
**E16 vs E18**	< 0.0001 $2*E^{-26}$	0.13262
**E18 vs P0/1**		0.66747
**P0/1 vs P2/3**		$3*E^{-6}$
**P2/3 vs P4/5**		0.00184
**P4/5 vs P6/7**		0.27361
**P6/7 vs P8/9**		0.00832
**P8/9 vs P10/11**		0.6884
**P10/11 vs P12/13**		0.04834
**P12/13 vs P14/15**		0.74245

One way ANOVA with Fisher's LSD post-hoc test

V_{thresh} (mV) groups	Univariate ANOVA between groups	Post-hoc test between the groups
**E16 vs E18**	< 0.0001 $5*E^{-27}$	0.00684
**E18 vs P0/1**		0.07704
**P0/1 vs P2/3**		$8*E^{-4}$
**P2/3 vs P4/5**		0.391
**P4/5 vs P6/7**		0.4119
**P6/7 vs P8/9**		0.04495
**P8/9 vs P10/11**		0.60781
**P10/11 vs P12/13**		0.25607
**P12/13 vs P14/15**		0.18016

One-way ANOVA Kruskal-Wallis test with Dunn's Multiple comparison post-hoc test

AHP (pA) groups	Kruskall-Wallis test p value	Dunn's adj. p value
**E16 vs E18**	< 0.0001	> 0,9999
**E18 vs P0/1**		> 0,9999
**P0/1 vs P2/3**		> 0,9999
**P2/3 vs P4/5**		> 0,9999
**P4/5 vs P6/7**		> 0,9999
**P6/7 vs P8/9**		> 0,9999
**P8/9 vs P10/11**		> 0,9999
**P10/11 vs P12/13**		> 0,9999
**P12/13 vs P14/15**		> 0,9999

Supplementary table 5

Statistical analysis (significant differences are highlighted in blue)

Supplementary Fig. 6 Development of the CGIN hyperpolarization-activated, cyclic nucleotide sensitive H current, I_h (E16-P15).

Supplementary Fig. 6b

One way ANOVA with Fisher's LSD post-hoc test

Groups	n (cells)	N (mice)	Amplitude of sag_{-100} Mean ± SEM
**P2/3**	22	7	2.31± 0.27
**P4/5**	20	6	2.01 ± 0.191
**P6/7**	17	4	1.31 ±0.06
**P8/9**	17	4	1.26 ±0.05
**P10/11**	12	4	1.14 ±0.023
**P12/13**	20	4	1.17 ± 0.02
**P14/15**	22	6	1.17 ±0.02

Amplitude of sag_{-100} groups	Univariate ANOVA between groups	Post-hoc test between the groups
**P2/3 vs P4/5**	$6*E^{-12}$	0.08529
**P4/5 vs P6/7**		$6*E^{-5}$
**P6/7 vs P8/9**		0.78918
**P8/9 vs P10/11**		0.49003

**P10/11 vs P12/13**		0.87603
**P12/13 vs P14/15**		0.988

Supplementary Fig. 6d,e

One-way ANOVA with Fisher's LSD post-hoc test

Groups	n (cells)	N (mice)	I_h amplitude Mean ± SEM	I_h density Mean ± SEM
**E16**	8	3	22.16 ± 1.92	109.59 ±23.83
**P1/0**	7	3	86.50 ± 29.22	226.36 ± 59.63
**P4/5**	12	3	157.79 ± 20.74	176.70 ± 30.50
**P7**	10	3	254.40 ± 17.40	63.58 ± 11.94
**P14/15**	11	3	224.64 ± 6.59	19.25 ±3.46

I_h amplitude groups	Univariate ANOVA between groups	Post-hoc test between the groups
**E16 vs P0/1**	$2*E^{-11}$	0.02598
**P0/1 vs P4/5**		0.00801
**P4/5 vs P6/7**		$2*E^{-4}$
**P6/7 vs P14/15**		0.21312

I_h density groups	Univariate ANOVA between groups	Post-hoc test between the groups
**E16 vs P0/1**	$6*E^{-12}$	0.01199
**P0/1 vs P4/5**		0.23127
**P4/5 vs P6/7**		0.00368
**P6/7 vs P14/15**		0.24453

Supplementary Fig. 6g

Paired sample 2-tailed t- test

Mean sag_{-40} amplitude groups	n (cells)	N (mice)	SAG_{-40} amplitude (mV) Mean ± SEM	Paired sample 2-tailed t - test
**Control P4/5**	5	3	1.34 ± 0.0884	0.00532
**ZD7288 P4/5**	5	3	1.06 ± 0.07	
**Control P14/15**	5	3	1.25 ± 0.03	$7*E^{-4}$
**ZD7288 P14/15**	5	3	1 ± 0	

Supplementary Fig. 6h

Paired sample 2-tailed t- test

Mean V_{Rest} groups	n (cells)	N (mice)	V_{Rest} Mean ± SEM	Paired sample 2-tailed t - test
**Control P4/5**	5	3	-60 ± 1.5	0.01722
**ZD7288 P4/5**	5	3	-67.4 ± 2.7	
**Control P14/15**	5	3	-59.48 ± 0.46	0.02304
**ZD7288 P14/15**	5	3	-70.6 ± 3.4	

Supplementary Fig. 6j

Paired sample 2-tailed t- test

Mean AP delay groups	n (cells)	N (mice)	AP Delay (Normalized to control) Mean ± SEM	Paired sample 2-tailed t - test
**Control P4/5**	5	3	1	0.04398
**ZD7288 P4/5**	5	3	2.12 ± 0.38	
**Control P14/15**	5	3	1	2*E-4
**ZD7288 P14/15**	5	3	2.12 ± 0.09	

Supplementary Fig. 6 k

Paired sample 2-tailed t- test

Mean maximal spike frequency groups	n (cells)	N (mice)	Mean maximal spike frequency (Normalized to control) Mean ± SEM	Paired sample 2-tailed t - test
**Control P4/5**	5	3	1	0.02583
**ZD7288 P4/5**	5	3	0.67 ± 0.09	
**Control P14/15**	5	3	1	0.00546
**ZD7288 P14/15**	5	3	0.62 ± 0.07	

Supplementary table 6

Statistical analysis (significant differences are highlighted in blue)

Supplementary Fig 5. Rates of morphological and electrophysiological CGINs parameters changes during development (semi-log plots) (E16-P15).

Supplementary Fig.5 p,q

Significance of differences between fast and slow slopes was calculated according to Graham Currell: Scientific Data Analysis. Excel analysis for Fig 3.1 http://ukcatalogue.oup.com/product/97... © Oxford University Press
https://www.youtube.com/watch?v=myL_qzuLHTQ

Parameters	Significance of differences between fast and slow slopes
Sholl area under curve	0.036
Critical_radius	-
Total dendritic length	0.0009
Number of dendritic nodes	$2*E^{-6}$
Ending_radius	-
Critical value (Nb of crossing intersections)	$4*E^{-5}$

Input Resistance	-
Vtresh	0.0005
AP amplitude	0.0002
AP duration	$7*E^{-5}$
Maximal frequency	$1*E^{-5}$
AHP	0.00014
SAG_{-100}	0.002
SAG_{-40}	0.0004

Supplementary table 7

Statistical analysis (significant differences are highlighted in blue)

Supplementary Fig. 7. Postnatal development of the CGIN persistent sodium current, I_{NaP} (P5-P15).

Supplementary Fig. 7b

One way ANOVA with Fisher's LSD post-hoc test

I_{NaP} peak amplitudes groups	n (cells)	N (mice)	I_{NaP} amplitude (pA) Mean ± SEM
**P4/5**	9	4	28.77 ± 3.05
**P7**	7	3	76.86 ± 16.87
**P14/15**	6	3	100.17 ± 11.30

Groups	Univariate ANOVA between groups	Post-hoc test between the groups
**P4/5 vs P7**	0.0044	0.0043
**P4/5 vs P14/15**		2*E-4
**P7 vs P14/15**		0.17085

Supplementary Fig. 7d

Two- sample 2-tailed t- test

Groups	n (cells)	N (mice)	Mean Frequency (Hz) Mean ± SEM	Two- sample 2-tailed t - test
**Control P7**	6	3	9.33 ±1.73	0.0010
**TTX P7**	6	3	1.17 ±.47	

Supplementary table 8

Statistical analysis (significant differences are highlighted in blue)

Figure 2. Developmental shift of $GABA_A$ polarity from excitatory to inhibitory

Figure 2 c

One way ANOVA with Fisher's LSD post-hoc test

Groups	n (cells)	N (mice)	Spike frequency (normalized to control) Mean ± SEM
**P0**	7	3	0.22 ± 0.06
**P2/3**	7	3	1.88 ± 0.17
**P4/5**	10	5	2.18 ± 0.22
**P6/7**	6	4	2.34 ± 0.36
**P8/9**	8	4	1.01 ± 0.42
**P10/11**	11	4	0.75 ± 0.195
**P12/13**	8	3	0.19 ± 0.07
**P14/15**	7	3	0.27 ± 0.09
**P30/40**	13	7	0.42 ± 0.08 (*)

Spike frequency (normalized to control) groups	Univariate ANOVA between groups	Post-hoc test between the groups
**P0 vs P2/3**	< 0.0001 $3*E^{-13}$	$3*E^{-6}$
**P2/3 vs P4/5**		0.31892
**P4/5 vs P6/7**		0.61796
**P6/7 vs P8/9**		$1*E^{-4}$
**P8/9 vs P10/11**		0.35129
**P10/11 vs P12/13**		0.05526
**P12/13 vs P14/15**		0.8177
**P14/15 vs P30/40**		0.58927

(*) Data from Lozovaya et.al. 2018

Supplementary table 9

Statistical analysis (significant differences are highlighted in blue)

Figure 3. Effects of blockers of $GABA_A$ synaptic transmission on CGINs ongoing activity before and after GABA polarity shift

Figure. 3 (a,b)

Two- sample 2-tailed t - test

groups	n (cells)	N (mice)	Spike frequency (Hz) Mean ± SEM	Two- sample 2-tailed t - test
**APV+CNQX P4/5**	12	6	1.74 ± 0.35	0.038
**APV+CNQX +Gabazine P4/5**	11	7	0.84 ± 0.20	
**APV+CNQX P14/15**	17	6	1.17 ± 0.27	0.59655
**APV+CNQX +Gabazine 14/15**	22	7	1.35 ± 0.18	

Fig. 3 (c)

Two- sample 2-tailed t - test

groups	n (cells)	N (mice)	Spike frequency (Hz)Mean ± SEM	Two-sample 2-tailed t - test
**Vehicle (DMSO) P4/5**	21	8	2.38 ± 0.60	0.031
**Bumetanide P4/5**	20	7	0.96 ± 0.16	

Supplementary table 10

Statistical analysis (significant differences are highlighted in blue)

Supplementary Fig. 9. Development of spontaneous $GABA_AR$-mediated activity recorded from CGINs (E18-P15).

Supplementary Fig. 9 (d-f)

One way ANOVA with Fisher's LSD post-hoc test

Groups	n (cells)	N (mice)	GABAergic PSCs Amplitude (pA) Mean ± SEM	GABAergic PSCs Frequency (Hz) Mean ± SEM	GABAergic PSCs Charge density Mean ± SEM
**E18**	9	5	43.12 ± 4.17	0.28 ± 0.07	0.33± 0.08
**P0**	9	5	46.16 ± 5.21	0.84 ± 0.36	0.68 ± 0.34
**P4/5**	10	8	36.44 ± 3.47	0.76 ± 0.15	0.64 ± 0.26
**P6/7**	9	4	37.65 ± 3.84	2.84 ± 0.70	2.55 ± 0.77
**P14/15**	9	5	36.56 ± 4.08	1.43 ± 0.53	0.91 ± 0.39

GABAergic PSCs Amplitude groups	Univariate ANOVA between groups	Post-hoc test between the groups
**E18 vs P0/1**	0.36352	0.61254
**P0/1 vs P4/5**		0.10186
**P4/5 vs P6/7**		0.83586
**P6/7 vs P14/15**		0.85586

GABAergic PSCs Frequency groups	Univariate ANOVA between groups	Post-hoc test between the groups
**E18 vs P0/1**	0.0014	0.36263
**P0/1 vs P4/5**		0.89262
**P4/5 vs P6/7**		0.001
**P6/7 vs P14/15**		0.0250

GABAergic PSCs Charge density groups	Univariate ANOVA between groups	Post-hoc test between the groups
**E18 vs P0/1**	< 0.0001<br>3*E^{-13}	0.5446
**P0/1 vs P4/5**		0.9426
**P4/5 vs P6/7**		0.002
**P6/7 vs P14/15**		0.00854

Supplementary table 11

Statistical analysis (significant differences are highlighted in blue)

Figure 4: Developmental shift from excitatory to inhibitory (pause), of the CGIN post train GABAergic response to cortical stimulation

Figure 4 e

One-way ANOVA Kruskal-Wallis test with Dunn's Multiple comparison post-hoc test

Groups	n (cells)	N (mice)	Spike frequency (normalized to baseline) Mean ± SEM
**Control P4/5 before train**	8	4	1.00 ± 0.025
**Control P4/5 500 ms after train**			2.91 ± 0.32
**Control P4/5 1400 ms after train**			1.55 ± 0.16
**Bumetanide P4/5 before train**	6	3	1.00 ± 0.03
**Bumetanide P4/5 500 ms after train**			0.06 ± 0.03
**Bumetanide P4/5 1400 ms after train**			0.030 ± 0.01

Groups	Kruskall-Wallis test p value	Dunn's adj. p value
**Control P4/5 before train vs Control P4/5 500 ms after train**	< 0,0001	< 0,0001
**Control P4/5 before train vs Control P4/5 1400 ms after train**		> 0,9999
**Control P4/5 500 ms after train vs Bumetanide P4/5 500 ms after train**		< 0,0001
**Control P4/5 1400 ms after train vs Bumetanide P4/5 1400 ms after train**		< 0,0001
**Bumetanide P4/5 before train vs Bumetanide P4/5 500 ms after train**		< 0,0001

**Bumetanide P4/5 before train vs Bumetanide P4/5 1400 ms after train**		< 0,0001

Figure 4 f

One-way ANOVA Kruskal-Wallis test with Dunn's Multiple comparison post-hoc test

Groups	n (cells)	N (mice)	Spike frequency (normalized to baseline) Mean ± SEM
**Control P4/5 before train**	8	4	1.00 ± 0.025
**Control P4/5 500 ms after train**			2.91 ± 0.32
**Control P4/5 1400 ms after train**			1.55 ± 0.16
**Control P12-14 before train**	6	3	0.99 ± 0.03
**Control P12-14 500 ms after train**			0.08 ± 0.05
**Control P12-14 1400 ms after train**			0.10 ± 0.02

Groups	Kruskall-Wallis test p value	Dunn's adj. p value
**Control P4/5 before train vs Control P4/5 500 ms after train**	< 0,0001	< 0,0001
**Control P4/5 before train vs Control P4/5 1400 ms after train**		> 0,9999
**Control P4/5 500 ms after train vs Control P 12-14 500 ms after train**		< 0,0001
**Control P4/5 1400 ms after train vs Control P12-14 1400 ms after train**		< 0,0001
**Control P12-14 before train vs Control P12-14 500 ms after train**		< 0,0001
**Control P12-14 before train vs Control P12-14 1400 ms after train**		< 0,0001

Supplementary table 12

Statistical analysis (significant differences are highlighted in blue)

Supplementary Fig. 12: Picrotoxin-sensitivity of the excitatory response of CGINs to cortical stimulation in striatal slices from control pups (P4-6).

Supplementary Fig. 12b

One-way ANOVA Kruskal-Wallis test with Dunn's Multiple comparison post-hoc test

Groups	n (cells)	N (mice)	Spike frequency (normalized to baseline) Mean ± SEM
**Control P4/5 before train**	8	4	1.00 ± 0.025
**Control P4/5 500 ms after train**			2.91 ± 0.32
**Control P4/5 1400 ms after train**			1.55 ± 0.16
**Picrotoxin P4/5 before train**	6	3	1 ± 0.02
**Picrotoxin P4/5 500 ms after train**			0.98 ± 0.11
**Picrotoxin P4/5 1400 ms after train**			0.80 ± 0.05

Groups	Kruskall-Wallis test p value	Dunn's adj. p value
**Control P4/5 before train vs Control P4/5 500 ms after train**	< 0,0001	< 0,0001

**Control P4/5 before train vs Control P4/5 1400 ms after train**		> 0,9999
**Control P4/5 500 ms after train vs Picrotoxin P4/5 500 ms after train**		< 0,0001
**Control P4/5 1400 ms after train vs Picrotoxin P4/5 1400 ms after train**		0.0003
**Picrotoxin P4/5 5 before train vs Picrotoxin P4/5 500 ms after train**		> 0,9999
**Picrotoxin P4/5 before train vs Picrotoxin P4/5 1400 ms after train**		0.0012

Supplementary table 13

Statistical analysis (significant differences are highlighted in blue)

Figure 5: Developmental shift from excitatory to inhibitory of the polysynaptic response between cholinergic interneurons

Fig. 5b

One way ANOVA with Fisher's LSD post-hoc test

Groups	n (cells)	N (mice)	Amplitude (pA) Mean ± SEM
**Control P4/5**	10	6	15.8 ± 3.9
**Nicotinic ant P4/5**	5	3	17.8 ± 5.9
**Control P7**	7	5	276.29 ± 49.42
**Nicotinic ant P7**	6	3	5.17 ± 1.99
**Control P14/15**	7	4	783 ± 196
**Nicotinic ant P14/15**	6	3	18.5 ± 12.9

Groups	Univariate ANOVA between groups	Post-hoc test between the groups
**Control P4/5 vs Nicotinic ant P4/5**	< 0.0001<br>$2*E^{-7}$	0.987
**Control P4/5 vs Control P7**		0.02316
**Control P7 vs Control P14/15**		$2E^{-4}$
**Control P7 vs Nicotinic ant P7**		0.0353
**Control P14/15 vs Nicotinic ant P14/15**		$5*E^{-7}$

Fig. 5e

Two-sample 2-tailed t - test

groups	n (cells)	N (mice)	PSP Onset (ms) Mean ± SEM	Two-sample 2-tailed t - test
**PSP onset P7**	5	3	22.5 3 ± 3.78	0.03
**PSP onset P14/15**	4	3	10.82 ± 0.66	

Fig. 5f

Two-sample 2-tailed t - test

groups	n (cells)	N (mice)	PSP Onset (ms) Mean ± SEM	Two-sample 2-tailed t - test
**PSP onset P7**	5	3	22.5 3 ± 3.78	0.012
**Delay between 1st and 2nd spikes**	6	4	39.13 ± 3.68	

Supplementary table 14

Statistical analysis (significant differences are highlighted in blue)

Supplementary Fig. 14: Age-dependent GABAergic component of the polysynaptic microcircuit from cortex to CGINs

Supplementary Fig. 14d

One way ANOVA with Fisher's LSD post-hoc test

groups	n (cells)	N (mice)	PSC amplitude (Normalized to control) Mean ± SEM
**Control P7**	9	3	1
**Nicotinic ant P7**			0.77 ± 0.04
**Control P14/15**	9	3	1
**Nicotinic ant P14/15**			0.42 ± 0.07

Groups	Univariate ANOVA between groups	Post-hoc test between the groups
**Control 7 vs Nicotinic ant P7**	< 0.0001 $3*E^{-11}$	$6*E^{-4}$
**Control P14/15 vs Nicotinic ant P14/15**		$4*E^{-11}$
**Nicotinic ant P7 vs Nicotinic ant P14/15**		$2*E^{-6}$

Supplementary table 15

Statistical analysis (significant differences are highlighted in blue)

Figure 6: Absence of developmental shift of the $GABA_AR$-mediated response and of cortico-striatal GABAergic pause response after early chronic treatment of pups with the NKCC1 blocker bumetanide

Fig. 6 e

One way ANOVA with Fisher's LSD post-hoc test

Groups	n (cells)	N (mice)	Mean frequency (Normalized to control) Mean ± SEM
**Control P13/14**	13	6	0.25 ± 0.06
**Bumetanide-I* P13**	16	3	1.00 ± 0.11
**Vehicle P13**	17	3	0.28 ± 0.06

*** Bumetanide-I - Bumetanide-treated mice**

Groups	Univariate ANOVA between groups	Post-hoc test between the groups
**Control P13/14 vs Bumetanide-I P13**	< 0.0001 $4*E^{-8}$	$3*E^{-7}$
**Control P13/14 vs Vehicle P13**		0.82
**Vehicle P13 vs Bumetanide-I P13**		$2*E^{-7}$

Fig. 6 f

One-way ANOVA Kruskal-Wallis test with Dunn's Multiple comparison post-hoc test

Groups	n (cells)	N (mice)	Spike frequency (Normalized to baseline) Mean ± SEM
**Control P12-14 before train**	6	3	0.99 ± 0.03
**Control P12-14 500 ms after train**			0.08 ± 0.05
**Control P12-14 1400 ms after train**			0.10 ± 0.02
**Bumetanide-I P12-14 before train**	7	3	1 ± 0.02
**Bumetanide-I P12-14 500 ms after train**			1.16 ± 0.13

**Bumetanide-I P12-14 1400 ms after train**			0.93 ± 0.06

Groups	Kruskall-Wallis test p value	Dunn's adj. p value
**Control P12-14 before train vs Control P12-14 500 ms after train**	< 0,0001	< 0,0001
**Control P12-14 before train vs Control P12-14 1400 ms after train**		< 0,0001
**Control P12-14 500 ms after train vs Bumetanide-I P12-14 500 ms after train**		< 0,0001
**Control P12-14 1400 ms after train vs Bumetanide-I P12-14 1400 ms after train**		< 0,0001
**Bumetanide-I P12-14 before train vs Bumetanide-I P12-14 500 ms after train**		> 0,9999
**Bumetanide-I P12-14 before train vs Bumetanide-I P12-14 1400 ms after train**		> 0,9999

Fig. 6 g

One-way ANOVA Kruskal-Wallis test with Dunn's Multiple comparison post-hoc test

Groups	n (cells)	N (mice)	Spike frequency (Normalized to baseline) Mean ± SEM
**Vehicle P12-14 before train**	10	3	1 ± 0.03
**Vehicle P12-14 500 ms after train**			0.06 ± 0.02
**Vehicle4 P12-14 1400 ms after train**			0.19 ± 0.03
**Bumetanide-I P12-14 before train**	7	3	1 ± 0.02
**Bumetanide-I P12-14 500 ms after train**			1.16 ± 0.13
**Bumetanide-I P12-14 1400 ms after train**			0.93 ± 0.06

Groups	Kruskall-Wallis test p value	Dunn's adj. p value
**Vehicle P12-14 before train vs Vehicle P12-14 500 ms after train**	< 0,0001	< 0,0001
**Vehicle P12-14 before train vs Vehicle P12-14 1400 ms after train**		< 0,0001
**Vehicle P12-14 500 ms after train vs Bumetanide-I P12-14 500 ms after train**		< 0,0001
**Vehicle P12-14 1400 ms after train vs Bumetanide-I P12-14 1400 ms after train**		< 0,0001
**Bumetanide-I P12-14 before train vs Bumetanide-I P12-14 500 ms after train**		> 0,9999
**Bumetanide-I P12-14 before train vs Bumetanide-I P12-14 1400 ms after train**		> 0,9999

SUPPLEMENTARY NOTE

MOTOR TESTS

Locomotor activity of P2 to P15 Lhx-6 GFP mice pups

*Locomotor and exploratory behavior in an open field (*40cm x 40cm*):* Each pup was placed in a clear enclosure where it was visible from the top as well as the side and observed for 3 min. We determined the age at which mice pups start to pivot, crawl or walk. Pivoting was defined as turning movements by broad swipes with forepaws, using only one hindlimb as a pivot and having the pelvis anchored to the ground. Crawling consisted of dragging the body forward or pushing it backward by undulating movements of the trunk and often dragging the hindlimbs in an extended position with foot soles facing upward. Walking appeared when the body was off the floor, supported by the four legs which moved in sequence. When the body was off the floor supported by the four legs but the hindlimbs were not moving in coordination with the forelimbs we defined it as transition from crawling to walking [1–4].

Huddling test was performed as described in [5] with some modifications. Huddling is a spontaneous, interactive, natural group-dependent behavior that manifests in many species, including mice pups. This behavior is associated with the formation and maintenance of active and prolonged contacts between 2 or more littermates. Litters were culled before P2/3 through random selection to yield a uniform litter size and sex composition of offspring, therefore overcoming litter size-dependent variabilities. For all tests, mice pups were transported from the animal facility to the experimental room at least 30 min before the start of the experiment for acclimatization. Tests were performed during the light period (between 12:00 and 15:00) under dim light illumination on a litter consisting of 9 pups and a dam. The littermates were separated from their mother and placed in an empty (40cm x 40cm) arena. The experiments were videotaped for 10 minutes by a camera placed on a bar above the apparatus using the Ethovision XT v14 software (Noldus). Videos were analyzed manually. The videos were viewed and scored by an investigator who was blind to the animal condition.

Ultrasonic vocalization (distress calls) of P2 to P15 Lhx-6 GFP mice pups

We recorded early isolation-induced ultrasonic vocalizations (USVs) from P2-P15 pups to evaluate the postnatal period at which this early communicative behavior declines and stops [6,7] and to identify the temporal coincidence with walking. We conducted testing in the morning, during the light cycle, in a room maintained at 21–22 °C. Following a 30-min habituation, pups were isolated one by one from their mother and placed into an isolation box (23 × 28 × 18 cm) located inside a sound-attenuated chamber (54 × 57 × 41 cm; Couldbourn Instruments, PA, USA). USVs were recorded for 3 min using an ultrasound microphone sensitive to frequencies of 10–250 kHz (Avisoft UltraSoundGate condenser microphone capsule CM16/CMPA; Avisoft bioacoustics, Germany) and the Avisoft recorder software (version 4.2) with a sampling rate of 250 kHz in a 16-bit format. Data were transferred to SASLab Pro software (version 5.2; Avisoft bioacoustics, Germany) and a fast Fourier transform (FFT) was conducted to create a spectrogram with a 512 FFT-length, 100% Hamming window, 75% temporal resolution overlap, frequency resolution of 488 Hz, and a sampling frequency of 22,050 Hz. Spectrograms were analyzed for the number of ultrasonic call [8].

Neurodevelopmental motor reflexes of P2 to P15 Lhx-6 GFP mice pups.

To investigate the development of motor reflexes in neonatal mice, we adapted to mice pups the SHIRPA (SmithKline Beecham, Harwell, Imperial College and Royal London Hospital Phenotype Assessment) neurological test battery originally developed in neurologically challenged adult rodents [2,4,9].

The surface Righting reflex is the motor ability for a pup to be able to flip onto its feet from a supine position. It is mediated by vestibular pathways, spinal interneurons, proprioceptive afferents, and motor neurons. There is no learning component in this test. Pups were placed on their backs on a cotton sheet and held in this position for 5 s and then released. We recorded the time each pup took to return to prone position. Pups that did not flip over in 30 s were given a zero score. Each pup underwent three trials. Pups begun to flip at P2/P3 and all mice were able to flip at P8 (Supplementary Fig. 1a**)**.

The Negative Geotaxis reflex is the ability for a pup, when placed facing down a slope, to turn to face up the slope. It reflects vestibular function (to recognize orientation on a slope) and motor coordination. Improvements on testing due to learning is possible. Pups were placed with their head pointing downward on a 45° incline, held for 5 sec and then released. Pups that fell down the incline or failed to turn in 30 s were given a zero score. Each pup underwent three trials. Pups begun to turn at P4 and all mice were able to face up the slope at P8 (Supplementary Fig. 1b**)**.

The cliff aversion reflex is the ability for a pup, when placed on the edge of a cliff with the forepaws and face over the edge, to turn and crawl away from the cliff drop. It tests labyrinth reflexes, as well as strength and coordination. Since cliff avoidance is not guided by visual information (pup's eyes are still closed), fear is not the driving factor to turn away from the cliff's edge. Improvements on testing due to learning is possible. Pups were be placed on the edge of a box (no less than 30 cm height) and the forepaws, digits and snout were the only parts over the edge. If no response is seen after 30 sec, the test was terminated. Each pup underwent three trials. Pups begun to avoid cliff at P4 and all mice were able to avoid it at P8 (Supplementary Fig. 1c**)**.

ELECTROPHYSIOLOGY and IMMUNOHISTOCHEMISTRY

Slice preparation. For prenatal dates E16 and E18, we euthanized pregnant dams by cervical dislocation, and rapidly (in 20–30 s) isolated uterine horns from their blood supply. We immediately delivered and decapitated pups. We removed fetuses from timed-pregnant females 72 ± 6h or 24 ± 6h before the estimated day of delivery, respectively. For postnatal dates P0 to P5 we quickly killed pups by decapitation but after P5 we anesthetized pups with isoflurane before decapitation. We kept brains in ice-cold oxygenated solution containing (in mM): 118 choline chloride, 2.5 KCl, 0.7 $CaCl_2$, 7 $MgCl_2$, 1.2 NaH_2PO_4, 26 $NaHCO_3$, 8 glucose, and performed parasagittal slices (300-350 µm thick) using a vibratome (VT1200 Leica Microsystems Germany). During the recovery period (at least 2h), slices were placed at room temperature (RT) in standard artificial cerebrospinal fluid (ACSF) saturated with 95% O_2 /5 % CO_2 and containing (in mM): 125 NaCl, 3.5 KCl, 0.5 $CaCl_2$, 3 $MgCl_2$, 1.25 NaH_2PO_4, 26 $NaHCO_3$ and 10 glucose, 300 $mOsmL^{-1}$. For recordings, we used an ACSF of the same composition but containing 2 mM $CaCl_2$ and 1 mM $MgCl_2$.

Patch-clamp recordings.

Slice preparation, patch clamp recordings, extracellular and optogenetic stimulations were performed as previously described [10]. Slices were transferred to the recording chamber and perfused with oxygenated ACSF (3 ml/min) at room temperature (22–25°C). Neurons were visualized using infrared-differential interference optics (Microscope Olympus BX51WI (Japan)). Patch pipettes were pulled from borosilicate glass capillaries (World Precision Instruments, Sarasota, USA).

Identification of recorded striatal neurons

In striatal slices from Lhx6-iCre;RCE-EGFP mice, we identified the dual cholinergic/GABAergic interneurons (CGINs), as EGFP+ neurons with a large soma (≥ 30 µm diameter), thick primary dendrites and a typical response to hyperpolarizing pulses. To confirm their cholinergic identity, we revealed the biocytin injected during whole cell recordings, and performed ChAT labelling. In striatal slices from ChAT-ChR2-EYFP mice we identified ChINs as EYFP+ neurons with a typical response to hyperpolarizing pulses. We EYFP was selectively expressed in 97.7±0.3% of $ChAT^+$ cells [10].

Immunohistochemistry and biocytin-filled cell revelation.

Striatal coronal slices were incubated with ChAT primary antibody coupled with a Donkey anti-Goat Alexa Fluor 633 (1:500, The Jackson Laboratory). After 20 to 24 hours fixation in 4% paraformaldehyde (Alfa Aesar™, Thermo Fisher Scientific Inc, USA) in phosphate-buffered saline (PBS, Gibco™, Thermo Fisher Scientific Inc, USA) at 4°C, slices were washed three times in PBS and after a 1 hour of preincubation in PBS containing 10% Normal Donkey Serum (NDS, Jackson ImmunoResearch Europe Ltd, UK), 1% Bovine Serum Albumine (BSA, sigma-Aldrich®, Merck KGaA, Germany) and 0.3% Triton X-100 (sigma-Aldrich®, Merck KGaA, Germany), they were incubated overnight at 4°C with Goat anti-ChAT antibody (AB144P, Chemicon®, Merck KGaA, Germany; 1 :300) in PBS containing 1% NDS, 1% BSA and 0.3% Triton X-100. The day after, slices were washed three times in PBS and incubated in PBS containing 1% BSA with a Donkey anti-Goat coupled to Alexa Fluor 555 (A-21432, Invitrogen™, Thermo Fisher Scientific Inc, USA; 1:500) and streptavidin coupled to Alexa Fluor 647 (S32357, Invitrogen™, Thermo Fisher Scientific Inc, USA; 1:500). Slices were then washed three

times in PBS, labelled with Hoechst solution (1:1000 in PBS), washed again and cover-slipped using Fluoromount-G™ mounting medium (Invitrogen™, Thermo Fisher Scientific Inc, USA).

Reconstruction of biocytin-filled neurons and Sholl analysis

For biocytin-filled cells localization, identification and reconstruction, confocal images were acquired on a SP8X Leica microscope (Leica Microsystems, Germany) using a diode 405 for the excitation of Hoechst or Alexa Fluor 405 (5%, spectral detection 415-460 nm), an OPSL 488 for the excitation of EGFP or Alexa Fluor 488 (10%, spectral detection 498-528 nm), an OPSL 552 for the excitation of Alexa Fluor 555 (5%, spectral detection 559-600 nm), and a diode 638 for the excitation of Alexa Fluor 647 (0.1%, spectral detection 649-776 nm). Images were acquired at 200 Hz using a 10x objective (for cells localization, pixel size = 2.2705 µm) and a 40x oil immersion objective (for cells identification and reconstruction, pixel size = 0.2838135 µm), pinhole set to "Airy 1", by scanning with a z step of 0.5016 µm when needed. Images were edited using Photoshop and Illustrator (Adobe Systems Software Ireland Ltd., Ireland). After images acquisition, stacks were imported in the open-source platform Fiji (https://fiji.sc/) and processed for neurons reconstruction and Sholl analysis ([10] and Supplementary methods). We considered for analysis identified CGINs and ChINs recorded from the dorsal striatum only.

SUPPLEMENTARY REFERENCES

1. Dehorter, N. *et al.* Onset of Pup Locomotion Coincides with Loss of NR2C/D-Mediated Cortico-Striatal EPSCs and Dampening of Striatal Network Immature Activity. *Front Cell Neurosci* **5**, 24 (2011).

2. Heyser, C. J. Assessment of Developmental Milestones in Rodents. *Curr. Protoc. Neurosci.* **25**, 8.18.1-8.18.15 (2003).

3. Dehorter, N., Vinay, L., Hammond, C. & Ben-Ari, Y. Timing of developmental sequences in different brain structures: Physiological and pathological implications. *Eur. J. Neurosci.* **35**, 1846–1856 (2012).

4. Feather-Schussler, D. N. & Ferguson, T. S. A Battery of Motor Tests in a Neonatal Mouse Model of Cerebral Palsy. *J. Vis. Exp.* **2016**, (2016).

5. Naskar, S. *et al.* The development of synaptic transmission is time-locked to early social behaviors in rats. *Nat. Commun.* **10**, 1–12 (2019).

6. Scattoni, M. L., Crawley, J. & Ricceri, L. Ultrasonic vocalizations: A tool for behavioural phenotyping of mouse models of neurodevelopmental disorders. *Neurosci. Biobehav. Rev.* **33**, 508–515 (2009).

7. Hofer, M. A., Shair, H. N. & Brunelli, S. A. Ultrasonic Vocalizations in Rat and Mouse Pups. *Curr. Protoc. Neurosci.* **17**, 8.14.1-8.14.16 (2001).

8. Chiesa, M. *et al.* Term or Preterm Cesarean Section Delivery Does Not Lead to Long-term Detrimental Consequences in Mice. *Cereb. Cortex* **29**, 2424–2436 (2019).

9. Hatcher, J. P. *et al.* Development of SHIRPA to characterise the phenotype of gene-targeted mice. *Behav. Brain Res.* **125**, 43–47 (2001).

10. Lozovaya, N. *et al.* GABAergic inhibition in dual-transmission cholinergic and GABAergic striatal interneurons is abolished in Parkinson disease. *Nat. Commun.* **9**, (2018).
