# Supplementary material for: The early excitatory action of striatal cholinergic-GABAergic microcircuits conditions the subsequent GABA inhibitory shift
Source: Commun Biol. 2023 Jul 14;6:723. doi: 10.1038/s42003-023-05068-7 (PMC10349145; doi:10.1038/s42003-023-05068-7)
Supplement: Supplementary file 2 — Description of Supplementary Data [file 42003_2023_5068_MOESM2_ESM.pdf]

Description of Additional Supplementary Files

File name: Supplementary Data

Description: The numerical source data for the graphs are available in Supplementary Data file with source data for Figs 1-6 (as separate tabs, grouped by experiment).
