# Supplementary material for: The early excitatory action of striatal cholinergic-GABAergic microcircuits conditions the subsequent GABA inhibitory shift
Source: Commun Biol. 2023 Jul 14;6:723. doi: 10.1038/s42003-023-05068-7 (PMC10349145; doi:10.1038/s42003-023-05068-7)
Supplement: Supplementary file 4 — Repoting Summary [file 42003_2023_5068_MOESM4_ESM.pdf]

nature portfolio

Corresponding author(s): Constance Hammond

Last updated by author(s): Jun 3, 2023

Reporting Summary

Nature Portfolio wishes to improve the reproducibility of the work that we publish. This form provides structure for consistency and transparency in reporting. For further information on Nature Portfolio policies, see our Editorial Policies and the Editorial Policy Checklist.

Statistics

For all statistical analyses, confirm that the following items are present in the figure legend, table legend, main text, or Methods section.

n/a	Confirmed	
☐	☒	The exact sample size (*n*) for each experimental group/condition, given as a discrete number and unit of measurement
☐	☒	A statement on whether measurements were taken from distinct samples or whether the same sample was measured repeatedly
☐	☒	The statistical test(s) used AND whether they are one- or two-sided<br>*Only common tests should be described solely by name; describe more complex techniques in the Methods section.*
☐	☒	A description of all covariates tested
☐	☒	A description of any assumptions or corrections, such as tests of normality and adjustment for multiple comparisons
☐	☒	A full description of the statistical parameters including central tendency (e.g. means) or other basic estimates (e.g. regression coefficient) AND variation (e.g. standard deviation) or associated estimates of uncertainty (e.g. confidence intervals)
☐	☒	For null hypothesis testing, the test statistic (e.g. *F*, *t*, *r*) with confidence intervals, effect sizes, degrees of freedom and *P* value noted<br>*Give P values as exact values whenever suitable.*
☒	☐	For Bayesian analysis, information on the choice of priors and Markov chain Monte Carlo settings
☒	☐	For hierarchical and complex designs, identification of the appropriate level for tests and full reporting of outcomes
☒	☐	Estimates of effect sizes (e.g. Cohen's *d*, Pearson's *r*), indicating how they were calculated

Our web collection on statistics for biologists contains articles on many of the points above.

Software and code

Policy information about availability of computer code

Data collection	Patch Master software (HEKA Elektronik, Germany), Andor iQ3 (Andor Technology, UK), Avisoft recorder software (version 4.2, Germany) were used for data collection
Data analysis	Prism 8 (GraphPad Software Inc., USA) , OriginPro 2019 (64-bit) 9.6.0.172 (OriginLab, USA), Clampfit 10.6.2.2 (Molecular Devices, LLC, USA) Igor 6.3.7.2 (WaveMetrics, Oregon, USA), SASLab Pro software (version 5.2; Avisoft bioacoustics, Germany) and open-source platform Fiji (https://fiji.sc/) softwares were used for data analysis.

For manuscripts utilizing custom algorithms or software that are central to the research but not yet described in published literature, software must be made available to editors and reviewers. We strongly encourage code deposition in a community repository (e.g. GitHub). See the Nature Portfolio guidelines for submitting code & software for further information.

Data

Policy information about availability of data

All manuscripts must include a data availability statement. This statement should provide the following information, where applicable:

- Accession codes, unique identifiers, or web links for publicly available datasets
- A description of any restrictions on data availability
- For clinical datasets or third party data, please ensure that the statement adheres to our policy

The numerical source data for the graphs are available in Supplementary Data file with source data for Figs 1-6 (grouped by experiment).

Human research participants

Policy information about studies involving human research participants and Sex and Gender in Research.

Reporting on sex and gender	N/A
Population characteristics	N/A
Recruitment	N/A
Ethics oversight	N/A

Note that full information on the approval of the study protocol must also be provided in the manuscript.

Field-specific reporting

Please select the one below that is the best fit for your research. If you are not sure, read the appropriate sections before making your selection.

☒ Life sciences ☐ Behavioural & social sciences ☐ Ecological, evolutionary & environmental sciences

For a reference copy of the document with all sections, see nature.com/documents/nr-reporting-summary-flat.pdf

Life sciences study design

All studies must disclose on these points even when the disclosure is negative.

Sample size	No sample size calculation was performed. Sample size was determined to be adequate based on the magnitude and consistency of measurable difference between groups. Sample size for animal studies in all sets was chosen from n> or = 50, number of litters n> or = 6
Data exclusions	The exclusion criteria were preestablished (see methods).
Replication	Replicate experiments were sucsessful
Randomization	The allocations of animals in groups was done randomly<br>Randomization was applied for morphological data set P4/5 group
Blinding	For behaviour studies the investigator was blind to the group allocation during analysis<br>Blinding was not relevant to invitro study because no bias could be made by the subject or the tester in the experiments performed.

Behavioural & social sciences study design

All studies must disclose on these points even when the disclosure is negative.

Study description	N/A
Research sample	N/A
Sampling strategy	N/A
Data collection	N/A
Timing	N/A
Data exclusions	N/A
Non-participation	N/A
Randomization	N/A

Ecological, evolutionary & environmental sciences study design

All studies must disclose on these points even when the disclosure is negative.

Study description	N/A
Research sample	N/A
Sampling strategy	N/A
Data collection	N/A
Timing and spatial scale	N/A
Data exclusions	N/A
Reproducibility	N/A
Randomization	N/A
Blinding	N/A

Did the study involve field work? ☐ Yes ☒ No

Reporting for specific materials, systems and methods

We require information from authors about some types of materials, experimental systems and methods used in many studies. Here, indicate whether each material, system or method listed is relevant to your study. If you are not sure if a list item applies to your research, read the appropriate section before selecting a response.

Materials & experimental systems

n/a	Involved in the study	
☐	☒	Antibodies
☒	☐	Eukaryotic cell lines
☒	☐	Palaeontology and archaeology
☐	☒	Animals and other organisms
☒	☐	Clinical data
☒	☐	Dual use research of concern

Methods

n/a	Involved in the study	
☒	☐	ChIP-seq
☒	☐	Flow cytometry
☒	☐	MRI-based neuroimaging

Antibodies

Antibodies used	Goat anti-ChAT antibody (AB144P, Chemicon®, Merck KGaA, Germany, Donkey anti-Goat coupled to Alexa Fluor 555 (A-21432, Invitrogen™, Thermo Fisher Scientific Inc, USA; 1:500),
Validation	All antibodies are commercially available and were commercially validated. All the available information on antibody validation can be found by searching the manufacturer's websites. All antibodies were validated by IHC in brins slices from Lhx6-iCre+/-;RCE-EGFP-/-mice (in addition see Lozovaya et al., Nat Commun. 2018 Apr 12;9(1):1422. doi: 10.1038/s41467-018-03802-y.)

Animals and other research organisms

Policy information about studies involving animals; ARRIVE guidelines recommended for reporting animal research, and Sex and Gender in Research

Laboratory animals	Following mice lines were used for the experiments: Lhx6-iCre+/-;RCE-EGFP-/- mice (generous gift from Prof. Gordon J. Fishell), ChAT-ChR2-EYFP mice (6.Cg-Tg(Chat-COP4*H134R/EYFP,Slc18a3)6Gfng/J, The Jackson Laboratory) and wild-type Swiss mice (CE Janvier, France)
Wild animals	Study did not involved a wild animals
Reporting on sex	Animals of both sex were taken for the experiments

Field-collected samples	N/A
Ethics oversight	Experiments were performed in agreement with the European community council directives (2010/63/UE). Protocols were approved by the local French ethical committee for animal experimentation (#19196) and validated by the French Ministry of Higher Education, Research and Innovation (authorization #19196-2018071214167976).

Note that full information on the approval of the study protocol must also be provided in the manuscript.
